# Rhizosphere Transformation and Plant Responses of Cerium Oxide Nanoparticles: Mechanistic Insights and Boundaries Compared with Other Rare-Earth Nanomaterials

**DOI:** 10.3390/plants15152354

**Published:** 2026-07-30

**Authors:** Xiaodan Wang, Shiwei Yuan, Yinghui Gu, Meiqi Pan, Xin Wang, Jianyi Wang, Xiuzhen Ni, Kai Song

**Affiliations:** 1 School of Life Science, Changchun Normal University, Changchun 130032, China; qx202510031@stu.ccsfu.edu.cn (X.W.); qx202510002@stu.ccsfu.edu.cn (S.Y.); qx202510003@stu.ccsfu.edu.cn (Y.G.); p2108635919@163.com (M.P.); wangxinresearch@163.com (X.W.); wjyhyjzxy@163.com (J.W.); 2 Institute of Innovation Science and Technology, Changchun Normal University, Changchun 130032, China

**Keywords:** cerium oxide nanoparticles, nano-ecotoxicology, plant toxicity, nanoceria, rhizosphere transformation

## Abstract

The increasing use of cerium oxide nanoparticles (CeO_2_-NPs) has raised concerns regarding their environmental fate and biological effects in soil–plant systems. This review uses cerium oxide nanoparticles (CeO_2_-NPs) as a mechanistically well-characterized case study to examine relationships among dose, chemical speciation, and plant responses, with emphasis on Ce^3+^/Ce^4+^ redox cycling and condition-dependent, nanozyme-like activity. We discuss how particle size, surface charge, Ce^3+^/Ce^4+^ ratio, and coating interact with rhizosphere processes, including organic acid complexation, phosphate-mediated CePO_4_ immobilization, and potentially microbially mediated transformation, to influence effective root-surface exposure. The resulting particulate, ionic, and secondary transformation-product pools affect root uptake, vascular transport, redox homeostasis, metabolism, and hormone-associated signaling. Major limitations include weak causal links between rhizosphere speciation and bioavailability, insufficient separation of particulate and ionic contributions, limited integration of multi-omics with speciation data, and uncertain extrapolation from hydroponic to soil systems. Standardized exposure protocols, integrated XANES, spICP-MS, and imaging workflows, and long-term soil studies are therefore required. Other rare-earth nanomaterials are discussed only briefly to define the material-specific boundaries of the CeO_2_-centered framework.

## 1. Introduction

The continued use of engineered nanoparticles (ENPs) in industrial and consumer products increases the likelihood of their release into soils, water bodies, and agricultural systems. In plants, particle transformation in the rhizosphere, the resulting effective exposure, and tissue accumulation are key processes linking environmental release to ecological and food safety risks. Rare-earth elements (REEs) usually refer to the lanthanides from La to Lu, together with Sc and Y, owing to their similar chemical properties. Owing to their unique 4f electronic configuration, REEs are widely used in communication technologies, electric vehicles, permanent magnets, lasers, catalysts, and green energy industries [1,2]. As REEs are increasingly used in functional materials, nanoscale rare-earth forms, including CeO_2_, La_2_O_3_, Y_2_O_3_, and upconversion nanoparticles (UCNPs), have also attracted attention in environmental and agricultural research [3,4].

Engineered CeO_2_-NPs can enter soil–plant systems through multiple pathways. Industrial wastewater discharge and atmospheric deposition may directly introduce particles into soils or water bodies. Nano-enabled fertilizers, pesticide formulations, and functional delivery systems may serve as agricultural exposure sources. Sewage sludge application and reclaimed-water irrigation may transfer particles or colloids enriched during wastewater treatment to farmland. In soils surrounding rare-earth mining areas and processing tailings, total REE concentrations may reach levels tens of times higher than background concentrations, creating distinct high-exposure scenarios [5,6,7,8]. It is important to distinguish high total Ce or REE concentrations in mining-affected soils from exposure to engineered CeO_2_-NPs. The particle forms in such soils may include mineral fragments, natural colloids, process-derived fine particles, and secondary precipitates. These input routes jointly shape the exposure scale and spatial distribution of rare-earth particles or colloids in agricultural ecosystems and provide the environmental context for studying plant effects.

Among published studies on plant responses to REE-NMs, CeO_2_-NPs are among the most frequently used materials for mechanistic investigation. Their value arises not only from their widespread industrial use and potential environmental exposure but also from their role as a representative redox-active rare-earth oxide. CeO_2_ has a cubic fluorite structure. Reversible surface Ce^3+^/Ce^4+^ conversion and oxygen vacancies can confer condition-dependent SOD-like, catalase-like, oxidase-like, or peroxidase-like activities [3]. These activities are not fixed material properties; rather, they depend on particle size, exposed crystal facets, surface coating, Ce^3+^ fraction, medium pH, substrate concentration, and biomolecular adsorption [4]. Therefore, CeO_2_-NPs are well suited for examining nonlinear dose–speciation–effect relationships. Their mechanisms, however, should not be directly extrapolated to La_2_O_3_, Y_2_O_3_, or UCNPs, for which dissolution, ion release, or optical tracing may be more relevant. Because CeO_2_-NPs are the representative material most frequently used for mechanistic analysis in this field, they are used here as the main focus of the discussion. A quantitative summary of their share in the literature is not attempted because the underlying figures are strongly dependent on the database, search terms, and time window, and no defensible primary statistical source is available.

This review aims to provide a mechanistically oriented synthesis of CeO_2_-NP behavior and effects in soil–plant systems, with four specific objectives. First, we critically examine how material descriptors—including particle size, surface charge, Ce^3+^/Ce^4+^ ratio, oxygen vacancies, and coating chemistry—interact with rhizosphere processes to govern aggregation, dissolution, complexation, and phosphate-mediated immobilization, thereby shaping effective root-surface exposure and internal transport. Second, we evaluate the dose-dependent and condition-specific nature of plant responses, distinguishing between ROS buffering and pro-oxidant outcomes and assessing the evidence for hormetic or biphasic patterns across species and exposure systems. Third, we identify critical knowledge gaps that currently limit causal interpretation, particularly the weak linkage between rhizosphere speciation and bioavailability, the insufficient separation of particulate and ionic contributions, and the limited integration of multi-omics with in situ elemental speciation data. Fourth, we propose standardized experimental frameworks and priority research directions—including combined XANES, spICP-MS, imaging workflows, long-term soil-based studies, and multi-omics–speciation integration—to improve cross-study comparability and strengthen the mechanistic basis for ecological and food-safety risk assessment. Throughout this review, CeO_2_-NPs are used as a mechanistically well-characterized case study; however, the Ce^3+^/Ce^4+^ redox cycling, oxygen-vacancy-mediated nanozyme-like activity, and Ce-specific phosphate transformation mechanisms discussed here should not be directly extrapolated to La_2_O_3_-NPs, Y_2_O_3_-NPs, or upconversion nanoparticles, which require material-specific evaluation.

Previous reviews have provided a valuable foundation for understanding plant responses to REE-NMs, but most have focused on individual toxicological endpoints, short-term hydroponic findings, or total-element dose–response relationships [9,10,11]. Less attention has been given to how rhizosphere transformations reshape particulate and ionic pools, how these pools determine bioavailability, and whether multi-omics responses can be causally linked to elemental speciation evidence. The limitations of extrapolating conclusions from hydroponic studies to soil and field conditions also remain insufficiently addressed. Concurrently, the biomedical field has advanced the use of CeO_2_-NPs as a promising nanotherapy for chronic degenerative diseases because of their regenerative redox activity [12], offering conceptual parallels that may inform plant stress-mitigation strategies.

To inform this narrative review, the Web of Science Core Collection, Scopus, and PubMed were searched for publications from January 2005 to April 2026. Search terms included combinations of “cerium oxide nanoparticles”, “CeO_2_ nanoparticles”, “nanoceria”, or “rare-earth nanomaterials” with “plant”, “rhizosphere”, “soil–plant”, “root uptake”, “phytotoxicity”, “bioavailability”, or “speciation”, together with parallel searches for La_2_O_3_, Y_2_O_3_, and upconversion nanoparticles. Peer-reviewed studies were included if they addressed the transformation, uptake, transport, bioavailability, or biological effects of CeO_2_-NPs in plant–rhizosphere systems. Selected studies on La_2_O_3_-NPs, Y_2_O_3_-NPs, and upconversion nanoparticles were retained only to define material-specific comparison boundaries. Biomedical and non-plant studies, as well as reports lacking sufficient material or exposure details, were excluded. Earlier seminal studies were retained where necessary to support foundational mechanisms. To clarify the strength of mechanistic interpretation, we distinguish throughout the review among directly supported, context-dependent, and tentative mechanisms. Mechanisms supported by direct speciation or imaging, controlled particle–ion or surface-coating comparisons, or functional perturbation in plant–rhizosphere systems are distinguished from mechanisms inferred primarily from correlations, omics associations, or analogies to other biological or material systems.

**Figure 1:**
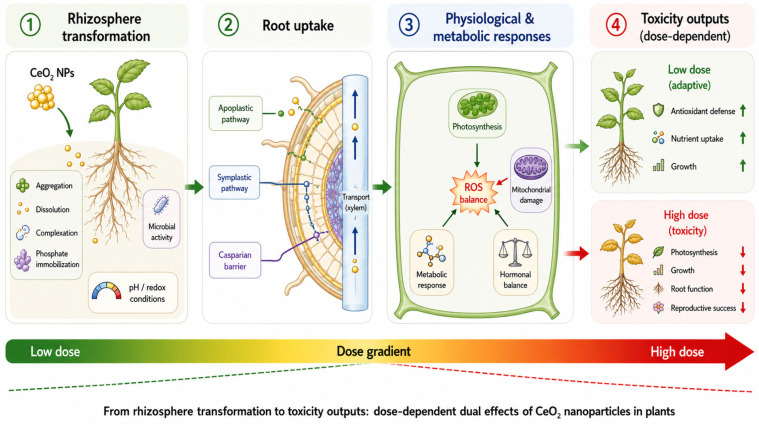
Dose-dependent rhizosphere transformation and plant responses of CeO_2_ nanoparticles. Arrows indicate the sequential processes from rhizosphere transformation to root uptake and plant responses; green and red arrows represent adaptive and toxic effects, respectively, while blue arrows indicate transport direction. Dashed lines indicate dose-dependent trends.

Across the available literature, reported plant responses to CeO_2_-NPs do not cluster clearly into a ‘predominantly beneficial’ or ‘predominantly adverse’ category. Low-dose exposures under favourable rhizosphere conditions have been repeatedly linked to ROS buffering, mild growth stimulation, and enhanced abiotic-stress tolerance, whereas higher doses, prolonged exposure, or rhizosphere conditions that promote surface Ce^3+^ release have more often been associated with oxidative damage, mineral-nutrient imbalance, and root-tip injury. We therefore treat CeO_2_-NP effects on plants as a condition-dependent bidirectional response rather than a fixed positive or negative outcome. Each major thematic section concludes with a brief synthesis of the principal findings, the limitations of the supporting evidence, and the remaining uncertainties. Figure 1 summarizes the conceptual framework adopted in this review, linking rhizosphere transformation and root uptake to downstream physiological, metabolic, and dose-dependent plant responses.

## 2. Physicochemical Properties and Transformation-Relevant Descriptors of CeO_2_ Nanoparticles

Sequential interaction chain from rhizosphere transformation to plant responses under CeO_2_-NP exposure. CeO_2_-NPs may undergo aggregation, sedimentation, dissolution, ligand exchange, phosphate-mediated immobilization, and potentially microbially mediated transformation in the rhizosphere. These processes regulate root-surface adsorption, cell-wall screening, vascular transport, and downstream redox and metabolic responses. The exposure gradient distinguishes possible adaptive responses at lower effective exposure from toxicological outcomes at higher effective exposure. The scheme is specific to CeO_2_-NPs and should not be generalized to other REE-NMs.

### 2.1. Crystal Structure, Ce^3+^/Ce^4+^ Cycling, and Oxygen-Vacancy-Associated Reactivity

CeO_2_-NPs generally possess a cubic fluorite crystal structure. The positions and broadening of X-ray diffraction peaks can be used to assess phase identity, crystallite size, and microstrain. However, the structural factors most relevant to plant-associated effects extend beyond the fluorite lattice itself. Exposed crystal facets, surface defects, oxygen-vacancy density, and the surface Ce^3+^/Ce^4+^ ratio jointly influence the direction and magnitude of ROS-related reactivity in the rhizosphere and plant-associated microenvironments.

Under defined experimental conditions, the coexistence of surface Ce^3+^ and Ce^4+^ species and oxygen-vacancy-associated defects can facilitate surface redox reactions and electron transfer. In cell-free or simplified assay systems, CeO_2_-NPs have displayed catalytic behaviors resembling those of superoxide dismutase, catalase, oxidase, or peroxidase [13,14]. However, the occurrence, direction, and apparent magnitude of these activities depend strongly on surface cerium speciation, exposed crystal facets, particle size and aggregation state, surface coatings, pH, ionic composition, substrate concentration, biomolecular adsorption, and the analytical methods used [15]. The relative contribution of each factor and the extent to which relationships identified in model systems apply to complex plant environments remain incompletely resolved.

**Figure 2:**
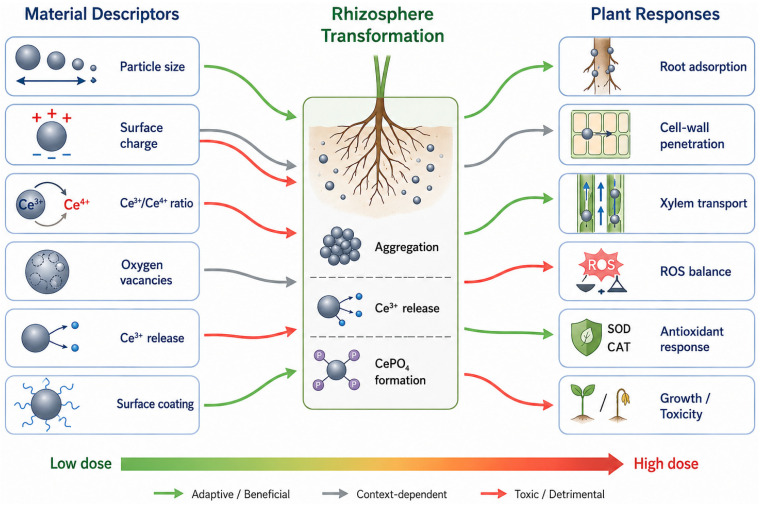
Material descriptors controlling CeO_2_-NP transformation and plant responses.

CeO_2_-NPs should therefore not be categorized a priori as either antioxidants or pro-oxidants. Their net ROS-related effects reflect the combined influence of particle transformation, local chemical conditions, biomolecular interactions, and endogenous ROS-producing and antioxidant processes in plants [16]. Evidence for direct intracellular nanozyme-like activity requires convergent information on Ce speciation, particle transformation, subcellular localization, and spatially and temporally resolved ROS dynamics. The strengths and limitations of this evidence are evaluated further in Section 6.1 (Figure 2).

### 2.2. Particle Size, Surface Charge, and Coating-Dependent Behavior

Beyond surface cerium speciation, the behavior of CeO_2_-NPs in plant exposure systems is strongly influenced by primary particle size, hydrodynamic diameter, aggregation state, surface electrokinetic properties, and coating chemistry. Primary size and morphology, typically characterized by transmission electron microscopy, affect specific surface area and the potential extent of particle–interface contact. In exposure media, however, aggregation, coating conformation, and adsorption of medium constituents can substantially alter hydrodynamic diameter and dispersion state. Root-surface association, therefore, depends on the combined effects of primary size, hydrodynamic size, aggregation state, coating thickness, and medium chemistry rather than on primary size alone [2,17,18].

Smaller primary particles may provide a larger specific surface area, but particle size should not be treated as a direct proxy for CeO_2_ surface reactivity or plant toxicity. Surface cerium speciation, exposed crystal facets, coating chemistry, and particle aging can modify the accessibility of reactive surface sites independently of primary size. Primary size and morphology should therefore be reported together with hydrodynamic diameter and aggregation state measured under relevant exposure conditions using dynamic light scattering or other appropriate methods.

Surface electrokinetic properties, commonly represented by ζ-potential, influence CeO_2_-NP aggregation and root-surface affinity but remain strongly dependent on exposure conditions. In cucumber (*Cucumis sativus*), positively charged chitosan-coated CeO_2_-NPs showed greater root retention than negatively charged poly(acrylic acid)-coated CeO_2_-NPs under the tested hydroponic conditions [19]. This comparison illustrates that coating-associated electrokinetic properties can affect root-surface interactions, but it should not be interpreted as a universal relationship between nominal particle charge and plant transport. ζ-Potential and colloidal behavior vary with pH, ionic strength, hydrodynamic size, coating conformation, and adsorption of root exudates or natural organic matter. The consequences of these differences for root-to-shoot transport are discussed in Section 4.6.

Coating chemistry further modifies dispersion stability, root-surface affinity, and the accessibility of CeO_2_ surface sites. Coated CeO_2_-NPs should therefore be characterized in the relevant exposure medium, and coating-matched controls should be included when the coating itself may influence plant responses. Particle size, surface electrokinetic properties, coating chemistry, and aging should be evaluated jointly rather than as independent predictors because their interactions determine the effective dispersion state and root-interface behavior of CeO_2_-NPs.

### 2.3. Dissolution, Ce^3+^ Release, and Transformation Propensity

Dissolution is a key process governing the distribution of Ce between particulate CeO_2_ and non-particulate Ce species and, consequently, the effective exposure of plant roots. CeO_2_-NPs are generally sparingly soluble under neutral and alkaline conditions. Nevertheless, partial surface dissolution may be enhanced in locally acidic, ligand-rich, or reducing microenvironments, where organic acids and other complexing agents can promote Ce^3+^ release. Such enhancement should be interpreted relative to neutral reference conditions and does not imply that CeO_2_-NPs are generally highly soluble in the rhizosphere [2,20,21,22,23,24].

Partial dissolution does not produce a simple binary mixture of intact particles and free ions. Intact or partially transformed CeO_2_ particles, dissolved Ce^3+^, Ce–organic ligand complexes, surface-adsorbed Ce species, and secondary solid phases such as CePO_4_ may coexist in exposure media, at root surfaces, and within plant tissues. Ce(III) should therefore not be treated as synonymous with freely dissolved Ce^3+^ because it may also occur in organic complexes, adsorbed forms, or Ce(III)-containing secondary phases. Conversely, the detection of Ce(IV) does not independently demonstrate the persistence of intact CeO_2_ nanoparticles. Nominal total-Ce concentration alone is consequently insufficient to characterize the biologically relevant exposure pool.

Resolving the relative contributions of these Ce pools requires convergent particle-sensitive, speciation-sensitive, and spatially resolved evidence supported by appropriate controls and total-Ce mass balance. The experimental controls used to distinguish particulate and non-particulate contributions are discussed in Section 4.2, whereas integrated analytical workflows and their methodological limitations are addressed in Section 9.3.

Taken together, dissolution, complexation, adsorption, and secondary-phase formation determine the relative abundance and potential bioavailability of different Ce pools. These processes are coupled to particle size, aggregation state, surface chemistry, and aging and should not be interpreted independently. Their extent and direction are governed by rhizosphere pH, organic ligands, phosphate availability, redox conditions, microbial activity, and exposure history, as discussed in Section 3.

### 2.4. Mechanistic Boundaries for Comparison with Other REE-NMs

Comparisons across rare-earth nanomaterial classes are most informative when restricted to shared exposure descriptors and biological response endpoints. Primary and hydrodynamic size, aggregation state, surface electrokinetic properties, coating chemistry, root-surface association, tissue distribution, growth responses, and oxidative-stress-related endpoints may provide useful comparative information. However, the occurrence of similar descriptors or phenotypic outcomes does not establish a common molecular mechanism.

In particular, the integrated CeO_2_ framework involving surface Ce^3+^/Ce^4+^ cycling, oxygen-vacancy-associated redox reactivity, and speciation among particulate CeO_2_, Ce(III)–organic complexes, and CePO_4_ should not be transferred wholesale to other REE-NMs. Other materials may share individual transformation processes or biological endpoints with CeO_2_-NPs, but their dominant mechanisms must be evaluated according to material-specific composition, dissolution behavior, lattice stability, and surface chemistry.

For La_2_O_3_-NPs, evidence from cucumber supports a dissolution–phosphate-reprecipitation pathway at the root nano–bio interface, in which partial dissolution may be followed by the formation of LaPO_4_-containing secondary phases [25]. Studies in other plant–exposure systems indicate that material form, nutrient disturbance, metabolic regulation, and biotic interactions may also influence La_2_O_3_-NP responses [26,27,28]. La_2_O_3_-NPs are therefore useful comparators for dissolution, ion release, and phosphate immobilization, but not for CeO_2_-specific redox cycling.

For Y_2_O_3_-NPs, the available plant evidence remains limited. Current observations support root-dominant accumulation of total Y and selected root biochemical responses but do not resolve whether intact Y_2_O_3_ particles, released Y^3+^, transformed Y-containing phases, or a combination of these forms is primarily responsible [29]. Y_2_O_3_-NPs can therefore be compared with CeO_2_-NPs at the levels of root retention and biological response endpoints, whereas their in planta chemical form and dominant transformation pathway remain unresolved.

The environmental and biological behavior of upconversion nanoparticles is strongly formulation-dependent. Host lattice, dopant composition, surface coating, colloidal stability, and exposure-medium chemistry jointly regulate particle persistence, ion release, plant-surface association, tissue localization, and biological effects [30,31]. UCNPs may therefore provide limited comparisons for coating-dependent stability, aggregation, ion release, and transport. Their optical functionality and lattice- and dopant-dependent behavior, however, place them outside the CeO_2_-specific redox framework.

Taken together, the most defensible comparisons across REE-NMs concern shared material descriptors, exposure processes, and biological endpoints rather than a unified molecular mechanism. La_2_O_3_-associated dissolution and phosphate reprecipitation, Y_2_O_3_ root accumulation with unresolved speciation, and formulation-dependent UCNP stability and transport should be evaluated separately from CeO_2_-associated redox and speciation processes. Because direct comparisons under matched plant species, medium chemistry, dose metrics, exposure duration, and analytical methods remain scarce, these distinctions should be regarded as evidence-based working boundaries rather than fixed or quantitatively ranked mechanistic categories.

In summary, material identity and physicochemical descriptors determine the initial potential of REE-NMs for aggregation, dissolution, surface reactivity, and root interaction, but their biological consequences cannot be predicted from any single descriptor. The roles of particle size, surface charge, coating, and CeO_2_-specific redox state are supported by experimental observations, whereas direct extrapolation across La_2_O_3_, Y_2_O_3_, CeO_2_, and UCNPs remains insufficiently validated. These material-specific differences provide the basis for understanding their subsequent transformation in the rhizosphere (Table 1).

## 3. Rhizosphere Transformation and Bioavailability Regulation

### 3.1. Chemical Complexity of the Rhizosphere

The rhizosphere is the microenvironment where plant roots exchange chemical, physical, and biological signals with soil. It is usually defined as the soil zone, at the millimeter scale, influenced by roots. Compared with bulk soil, the rhizosphere is highly heterogeneous in several ways. Roots continuously release low-molecular-weight organic acids, such as citrate, oxalate, malate, and tartrate, as well as amino acids, sugars, and phenolic compounds. Their concentrations can be much higher than those in bulk soil. Selective ion uptake by roots can cause local rhizosphere pH to deviate from bulk soil pH [35]. Microbial biomass and metabolic activity are usually higher in the rhizosphere than in non-rhizosphere soil. Oxygen concentration and electron donor/acceptor distribution near the root surface are uneven and may generate local reducing microzones [36,37,38,39,40]. These gradients are not fixed. They vary with plant species, growth stage, soil buffering capacity, and moisture status.

These gradients mean that particles near the root surface often experience a chemical environment different from that in a homogeneous hydroponic solution. The nominal addition concentration therefore describes only the external dose applied to the system. It cannot directly represent the effective dose at the root surface. Rhizosphere transformation links external exposure to internal responses. It is a key process for explaining differences in plant effects and remains relatively underdeveloped in current research.

### 3.2. Aggregation, Sedimentation, and Dissolution Kinetics

**Figure 3:**
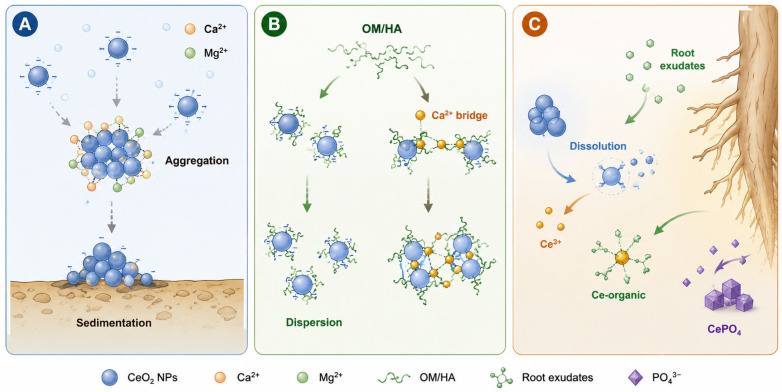
Rhizosphere controls on CeO_2_-NP transformation. Ca^2+^ and Mg^2+^ promote aggregation (**A**); organic matter can stabilize particles or induce cation bridging (**B**); root exudates and phosphate regulate dissolution, complexation, and CePO_4_ formation (**C**). Arrows indicate the direction of aggregation and transformation processes.

After entering the rhizosphere, CeO_2_-NPs may simultaneously undergo aggregation, sedimentation, surface ligand exchange, redox transformation, and local dissolution [41]. In rhizosphere solutions with relatively high concentrations of divalent cations such as Ca^2+^ and Mg^2+^, the electric double layer at the particle surface is compressed. The electrostatic repulsion barrier decreases, and CeO_2_-NPs are more likely to shift from a dispersed state to aggregates and then sediment (Figure 3A). Aggregate formation often reduces effective surface area and accessibility of surface-active sites. It can weaken interfacial reactivity and limit passage through cell-wall pores or root-surface micropores [2,42,43]. However, the effect of aggregation on bioavailability depends on the exposure system. In hydroponic systems, sedimented aggregates may cover the root surface and increase local external exposure. In soil pores, aggregation is more likely to reduce long-distance transport.

The effect of organic matter on CeO_2_-NPs depends on the ionic background (Figure 3B). Under low ionic strength, carboxyl and phenolic hydroxyl groups in humic acid or root exudates may improve particle dispersion through charge regulation and steric stabilization. When Ca^2+^ or Mg^2+^ is abundant, the same organic matter may promote aggregation and sedimentation through cation bridging. This bidirectional regulation depends on the type and concentration of organic matter, ionic strength, and pH. The biological consequences of organic-matter coating are also dual. Improved colloidal stability may enhance particle migration and accumulation at the root surface. At the same time, the coating layer may shield active Ce^3+^ sites and reduce direct toxicity or nanozyme-like activity. Therefore, plant responses should be interpreted by considering two opposing effects: enhanced mobility and reduced interfacial reactivity. In addition to influencing aggregation, root-derived low-molecular-weight organic acids can directly participate in surface complexation, local reduction, and dissolution of CeO_2_-NPs. Citrate, malate, succinate, and related ligands can interact with surface Ce sites, promote partial dissolution, and release Ce^3+^. The released Ce^3+^ can form Ce-organic acid complexes or, under phosphate-rich conditions, precipitate or become immobilized as CePO_4_ (Figure 3C) [2,44,45]. Different organic acids do not act in the same direction. Citrate and malate often favor complexation and migration, whereas oxalate may promote complexation but can also form low-solubility precipitates or surface complexes. Thus, the actual bioavailability of CeO_2_-NPs in the rhizosphere is not determined by nominal exposure concentration alone. It is jointly regulated by ionic strength, organic matter/organic acids, pH, phosphate, and the root-surface nano–bio interface. Thus, organic matter and root-derived ligands can simultaneously alter particle mobility and surface reactivity, and their net effect on bioavailability depends on ionic strength, ligand identity, pH, phosphate, and divalent-cation concentrations.

### 3.3. Phosphate Ligand Regulation: CePO_4_ Immobilization and Transport Balance

Phosphate is an important ligand regulating the rhizosphere fate of CeO_2_-NPs and Ce-containing transformation products. Phosphate (PO_4_^3−^) has a strong affinity for Ce^3+^. The solubility product of CePO_4_ is generally very low, allowing dissolved Ce^3+^ to precipitate as CePO_4_ microcrystals under suitable conditions and to become immobilized at the root surface or in the rhizosphere, thereby reducing bioavailability [32]. Synchrotron μ-XANES data from split-root cucumber experiments showed clear CePO_4_ signals near the root surface. Under sufficient phosphorus supply (+P), the shoot translocation factor of Ce was markedly lower than under phosphorus deficiency (−P) [32]. Under phosphorus deficiency, soluble Ce-carboxylate complexes in the rhizosphere increased, and Ce migration to shoots became more efficient. These findings show that phosphate supply can substantially alter CeO_2_-NP transformation and transport in controlled systems. Whether the same effect increases bioavailability in real phosphorus-deficient soils also depends on soil pH, Fe/Al oxides, clay minerals, organic matter, and rhizosphere buffering capacity.

These findings also have methodological implications. In hydroponic exposure experiments, if nutrient-solution phosphate concentration, Ce^3+^ activity, and pH differ greatly from those in real soil pore water, the reliability of extrapolating hydroponic results to soil exposure risk will decrease.

### 3.4. Complexation by Organic Acids and Root Exudates

Low-molecular-weight organic acids (LMWOAs) are major components of root exudates and important chemical factors controlling Ce speciation during CeO_2_-NP transformation in the rhizosphere. Citrate, oxalate, malate, and related LMWOAs can complex Ce^3+^ and form Ce–carboxylate complexes near the root surface. Under certain conditions, these complexes can compete with CePO_4_ precipitation [2,33]. However, the effect of organic acids on Ce migration depends on the type and concentration of the organic acid, pH, phosphate level, and Ca^2+^ bridging. It should not be described as a unidirectional enhancement. Organic acid secretion also varies according to root developmental stage, nutritional stress, and plant species. For example, phosphorus deficiency can induce strong citrate release, increase rhizosphere Ce–citrate concentrations, and compete with phosphate immobilization.

Root exudate composition differs substantially among plant species. This variation may be an important rhizosphere-chemical factor contributing to differences in plant responses at the same nominal exposure concentration. Therefore, rhizosphere chemistry should be considered when comparing toxicity data across species.

### 3.5. pH Gradients, Redox Status, and Microbially Mediated Transformation

Rhizosphere pH gradients strongly affect CeO_2_-NP dissolution, the Ce^3+^/Ce^4+^ ratio, and CePO_4_ precipitation. When NH_4_^+^ is the main nitrogen source, many plant roots acidify the rhizosphere, which may favor local dissolution of CeO_2_-NPs and Ce^3+^ release. When NO_3_^−^ is the main nitrogen source, the rhizosphere often becomes relatively alkaline, which may favor Ce^3+^ immobilization or CePO_4_ precipitation [22,23,32,35,46,47]. These trends also depend on plant species, rhizosphere buffering capacity, phosphate supply, and organic acid secretion.

Local reducing microzones in the rhizosphere (low-Eh regions) can promote partial conversion of Ce(IV) to Ce(III), thereby increasing dissolution [41]. Under alternating redox conditions in intermittently flooded rice (*Oryza sativa*) fields, reducing phases may partially dissolve CeO_2_-NPs and temporarily increase Ce mobility. Subsequent re-oxidation may promote the re-oxidation of Ce^3+^, phosphate precipitation, or binding to Fe/Mn oxides [24]. This dynamic redox process is important for assessing CeO_2_-NP bioavailability in paddy ecosystems and is difficult to capture in static hydroponic experiments.

Rhizosphere microorganisms may influence CeO_2_-NP fate through several pathways. Acid-producing bacteria may promote particle dissolution by lowering local pH. Extracellular polymeric substances (EPS) may adsorb or immobilize particles. Iron-reducing bacteria, such as *Geobacter* and *Shewanella*, could theoretically contribute to Ce(IV) reduction to Ce(III), but direct evidence for their quantitative contribution to CeO_2_-NP transformation in real rhizospheres is lacking. Phosphate-solubilizing bacteria and phosphatase-producing microorganisms can also exert bidirectional effects. Organic acid secretion may promote Ce dissolution and complexation-driven migration, whereas phosphorus release may enhance CePO_4_ immobilization. The quantitative contribution of these microbial processes has not been sufficiently validated and represents an important research gap.

### 3.6. Particle Aging and Long-Term Speciation Evolution

Long-term aging of CeO_2_-NPs in soil can affect risk-assessment outcomes (Table 2). Particle forms observed in short-term exposure experiments may not represent the actual forms present after weeks or months of aging. Available studies show that after CeO_2_-NPs have aged in soil for several weeks, a portion of the Ce may transform into CePO_4_ or co-precipitate with Fe/Mn oxides, reducing bioavailability. Newly added particles often show higher bioavailability [24]. However, aging does not always reduce risk uniformly. In systems with organic-matter coatings, colloidal stabilization, or periodic reduction–oxidation, Ce may show episodic release or pulse-like migration. Therefore, short-term hydroponic experiments using fresh particles may overestimate long-term soil exposure risk, but they may also fail to capture brief periods of high bioavailability under redox fluctuations. Aging time and redox history should be treated as key parameters in future risk assessments.

In summary, rhizosphere pH, phosphate, organic ligands, divalent cations, redox conditions, and microbial activity jointly regulate the aggregation, dissolution, complexation, and immobilization of CeO_2_-NPs. Phosphate-mediated CePO_4_ formation and ligand-dependent transformation are supported under controlled experimental conditions, whereas the quantitative contribution of microbially mediated transformation in real rhizospheres remains uncertain. These processes determine effective exposure at the root surface and therefore influence subsequent uptake and transport.

## 4. Root Uptake, Barrier Retention, and Internal Plant Transport

### 4.1. Root-Surface Adsorption and Cell-Wall Screening

The root surface is the first physical interface between CeO_2_-NPs and plants and is a key control point for subsequent internal exposure. The root cell wall is a negatively charged porous network composed of cellulose, hemicellulose, and pectin. It can retain nanoparticles through electrostatic adsorption, hydrogen bonding, ligand complexation, and physical interception [2,17,18]. Particle binding strength at the root surface depends on surface charge (ζ-potential), particle size, coating type, and medium ionic strength. Positively charged particles, such as CS-nCeO_2_, strongly adsorb onto negatively charged root surfaces and form dense surface-bound layers. Their translocation factors are often lower than those of negatively charged particles at the same dose, such as PAA-nCeO_2_ [19].

Physical screening by the cell wall is a key step in controlling internal exposure. Particles with hydrodynamic sizes smaller than the effective cell-wall pore size, usually estimated at 5–20 nm but variable with hydration status and pectin-network conformation, may pass through the cell wall into the apoplast and cortex. Larger particles or aggregates are more likely to adsorb to and remain at the root surface. They may form particle-accumulation layers and cause local ionic exposure through Ce^3+^ release. Therefore, cell-wall adsorption is not equivalent to harmless retention. It may also serve as a site of particle dissolution, speciation transformation, and local toxicity.

### 4.2. Distinguishing Particulate and Ionic Contributions

Distinguishing particulate CeO_2_ from dissolved Ce species is essential for identifying the dominant uptake and toxicity pathways in plants. Total Ce concentration alone cannot resolve these contributions because intact particles, dissolved Ce^3+^, Ce–organic ligand complexes, and secondary transformation products such as CePO_4_ may coexist in the exposure medium, at the root surface, and within plant tissues. In particular, Ce(III) should not be treated as synonymous with dissolved ionic Ce, because Ce(III) may also occur in organic complexes, adsorbed forms, or solid secondary phases.

Matched particle–ion controls provide the most direct experimental comparison. CeO_2_-NP treatments can be compared with soluble Ce salts at equivalent total Ce concentrations to determine whether the ionic treatment reproduces the observed uptake or biological responses. However, soluble Ce controls do not necessarily represent free Ce^3+^ activity because hydrolysis, ligand complexation, and phosphate precipitation may occur in nutrient solutions and rhizosphere environments. Dissolution-supernatant controls, dialysis, or ultrafiltration can therefore be used to estimate the contribution of the dissolved or low-molecular-weight fraction. These approaches provide an operational rather than absolute separation because small colloids may pass through membranes and dissolved Ce may adsorb onto filtration materials.

Single-particle ICP-MS can provide particle-sensitive information by distinguishing discrete particle-associated signals from the continuous dissolved Ce background and can be used to estimate particle number and particle-equivalent size. Its application to plant tissues requires cautious sample preparation because extraction, aggregation, and particle transformation may alter the original particulate fraction. The results should therefore be interpreted together with total-Ce recovery and mass-balance information rather than as independent proof of particulate uptake.

XANES and EXAFS provide complementary information on the Ce oxidation state and local chemical environment and can support the identification of CeO_2_, Ce(III)-organic complexes, and CePO_4_ when suitable reference materials are included [32,33,34,49]. However, oxidation state is not equivalent to physical state. A Ce(IV) signal does not independently prove the presence of intact CeO_2_ nanoparticles, and a Ce(III) signal does not distinguish dissolved Ce^3+^ from Ce(III)-containing complexes or solid CePO_4_. Similarly, μ-XRF and TEM–EDS can reveal the spatial distribution and particle-associated localization of Ce, but they cannot independently quantify the ionic contribution.

Accordingly, particulate and ionic contributions should be considered adequately distinguished only when matched particle–ion controls, operational size separation, particle-sensitive measurements, chemical-speciation analysis, and total-Ce mass balance provide mutually consistent evidence. Measurements of pH, phosphate, dissolved organic carbon, and relevant organic ligands are also necessary because these factors regulate CeO_2_ dissolution, Ce^3+^ complexation, and CePO_4_ formation.

### 4.3. Parallel Apoplastic and Symplastic Pathways

After cell-wall screening, CeO_2_-NPs or their transformation products may move inward through apoplastic and potentially symplastic pathways. The apoplastic pathway uses cell-wall and intercellular spaces as transport channels. Particles can move through the root cortex without crossing the plasma membrane, but this pathway is blocked at the endodermis by the Casparian strip and cannot directly enter the mature stele. The symplastic pathway requires particles or ions to cross the plasma membrane into the cytoplasm and then move through plasmodesmata in the cellular continuum. Intact nanoparticles must overcome the plasma-membrane barrier to use this pathway. Endocytosis-like mechanisms may be involved, but direct evidence in CeO_2_-NP–plant systems remains limited. This pathway is strongly constrained by particle size, hydrodynamic diameter, surface hydrophilicity, and coating state [17,44,50]. Even if intact CeO_2_-NPs do not efficiently cross the plasma membrane, their accumulation at the root surface and in the apoplast can still alter internal exposure through local ROS production, surface transformation, and limited dissolution. Released Ce^3+^ or Ce-organic ligand complexes may enter the symplast through non-selective cation channels or metal transport-related pathways. However, Ca^2+^ channels, NRAMP family members, CNGC/GLR channels, and related proteins should currently be regarded as candidate mechanisms rather than verified pathways. Future work should combine channel inhibitors, mutants, transporter expression analysis, free Ce^3+^ activity measurements, and elemental speciation tracing to separate ionic transmembrane transport from apoplastic particle migration. Overall, available evidence more strongly supports apoplastic retention and transformation than direct symplastic transport of intact CeO_2_-NPs, while the molecular pathways responsible for ionic Ce uptake remain to be validated.

### 4.4. Casparian Strip Retention and Developmental Windows

The Casparian strip is a lignin- and suberin-rich band located within the radial and transverse walls of endodermal cells. It forms a physicochemical barrier that blocks nonselective apoplastic transport into the stele. Its retention efficiency for particulate CeO_2_ and Ce-containing secondary phases depends on its developmental maturity. Near the root elongation zone, the Casparian strip is not fully established. Particles or soluble transformation products may pass through this immature region to approach the stele via the apoplastic pathway. As the distance from the root tip increases, the Casparian strip undergoes progressive secondary thickening, thereby increasing its barrier efficiency.

This developmental pattern has physiological significance. The root elongation zone may provide a spatial and temporal window for particulate CeO_2_ or mobile Ce-containing transformation products to approach the stele. It is also a highly sensitive toxicological target because cell division, elongation, and cell-wall remodeling are active in this region. Early root-tip damage may result from particle intrusion, local Ce^3+^ release, ROS accumulation, inhibited cell-wall remodeling, and suppressed cell division. Understanding this window effect may help predict transport efficiency and toxicological targets at different growth stages.

### 4.5. Transport Partitioning Between Particulate and Ionic Forms

Particulate CeO_2_-NPs and dissolved Ce^3+^ have different transport routes and kinetics within plants. Particulate Ce mainly accumulates in the root cortex via the apoplast. Most particles are retained at the Casparian strip, and only a small fraction may enter the stele. Ce^3+^ is smaller and, under certain coordination environments, may chemically compete with cations such as Ca^2+^. It may enter the stele through non-selective cation transport systems or metal transporters. Its xylem loading efficiency is usually higher than that of intact particles [17,44,50]. However, a similar ionic radius alone is not sufficient to demonstrate that Ce^3+^ and Ca^2+^ share the same transport channels, because Ce^3+^ and Ca^2+^ differ in valence, hydrated radius, coordination number, and ligand affinity.

This partitioning leads to different spatial distributions of CeO_2_-NPs and CeCl_3_ within plants. CeO_2_-NPs more often exhibit particulate accumulation and local effects in roots, especially in the cortex. CeCl_3_ is more likely to cause systemic Ce^3+^ translocation and whole-plant effects. This partitioning may help explain variations in shoot/root Ce concentration ratios, or translocation factors, among studies. However, few studies have systematically distinguished the relative contributions of these two transport routes.

### 4.6. Xylem Transport, Phloem Redistribution, and Internal Reallocation

**Figure 4:**
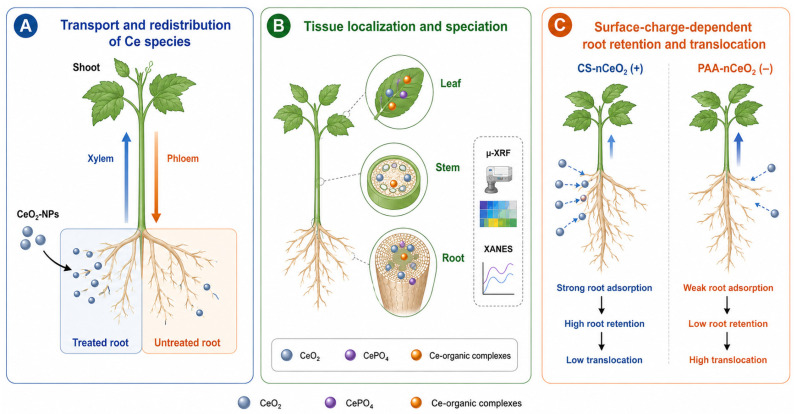
Transport, Redistribution, and Speciation of CeO_2_-Derived Ce in Plants. (**A**) Xylem transport from treated roots to shoots and phloem redistribution to untreated roots; (**B**) tissue localization and speciation of Ce in roots, stems, and leaves; (**C**) surface-charge-dependent root retention and translocation of CS-nCeO_2_ and PAA-nCeO_2_. Blue, purple, and orange spheres represent CeO_2_, CePO_4_, and Ce–organic complexes, respectively. Dashed lines indicate enlarged tissue regions or root adsorption, while arrows indicate transport direction; blue and orange arrows in (**A**) represent xylem and phloem transport, respectively.

Split-root experiments in cucumber provide evidence that Ce derived from CeO_2_-NP exposure can move from treated roots to shoots and subsequently be redistributed to untreated roots, a pattern consistent with xylem-mediated upward transport followed by phloem-mediated reallocation (Figure 4A) [34]. Treated roots and shoots contained mixtures of Ce(III)- and Ce(IV)-containing species, whereas Ce in untreated roots was predominantly detected as Ce(IV). These observations support the long-distance transport of Ce-containing species but do not identify the dominant physical or chemical form during translocation. In particular, the detection of Ce(IV) in untreated roots does not demonstrate that Ce remained as intact CeO_2_ throughout phloem transport, because reoxidation, deposition, or tissue-level transformation may have occurred after translocation [34]. Long-distance movement should therefore be interpreted as a dynamic, speciation-dependent process rather than as exclusively particulate or ionic transport.

μ-XRF mapping has localized Ce preferentially at root tips, lateral-root initiation zones, leaf veins, and leaf margins, consistent with vascular association and, at leaf margins, transpiration-associated deposition (Figure 4B) [19,34]. However, μ-XRF provides elemental localization and cannot by itself determine whether the detected Ce is present as intact CeO_2_ nanoparticles. Complementary XANES analyses indicate that root Ce may occur as mixtures of CeO_2_ and CePO_4_, whereas shoot Ce may include CeO_2_ and Ce-carboxylate-associated forms [19,33,34]. Together, these observations suggest that CeO_2_-NPs can undergo partial dissolution, reduction, phosphate precipitation, and organic-ligand complexation during uptake and internal transport. Elemental mapping should therefore be interpreted together with speciation-sensitive analyses when assigning transport forms.

Surface properties further regulate the balance between root retention and shoot translocation (Figure 4C). In cucumber, positively charged chitosan-coated CeO_2_ nanoparticles (CS-nCeO_2_) showed stronger root-surface adsorption, greater root Ce accumulation, and lower root-to-shoot translocation factors than negatively charged poly(acrylic acid)-coated CeO_2_ nanoparticles (PAA-nCeO_2_) [19]. PAA-nCeO_2_ was less strongly retained at the root surface and showed relatively greater shoot translocation. These comparisons show that coating-dependent surface charge and colloidal behavior modulate root retention and apparent translocation efficiency, although the magnitude of these effects also depends on exposure-medium chemistry, root exudates, ionic strength, and particle aging.

Overall, available evidence supports vascular transport and internal redistribution of a fraction of Ce derived from CeO_2_-NP exposure. However, the relative contributions and interconversion of intact CeO_2_ particles, dissolved or organically complexed Ce(III), and secondary Ce phases remain unresolved.

In summary, root-surface adsorption, cell-wall screening, and endodermal barriers result in the predominant retention of CeO_2_-derived Ce in roots. A smaller fraction undergoes vascular transport and internal redistribution, but the relative contributions and interconversion of particulate CeO_2_, dissolved or complexed Ce(III), and secondary Ce phases remain incompletely resolved. Quantitative separation of these pools is therefore required to link internal Ce exposure with downstream physiological responses.

## 5. Plant Morphological and Physiological Responses

### 5.1. Seed Germination and Early Radicle Development

Seed germination is one of the stages of the plant life cycle most sensitive to exogenous chemical exposure. During this stage, seed-coat hydration, radicle elongation, and cell division can be affected by particles or ions. Available studies on CeO_2_-NPs show that low doses can increase germination percentage, germination vigor, and germination rate in some systems and promote early radicle elongation [51]. In contrast, high doses or Ce^3+^ treatments at an equivalent Ce concentration may delay germination, reduce the germination index, and impair early cell division in the root apical meristem [1,2,34,52]. Hydroponic mg/L doses cannot be directly converted to soil mg/kg doses. Many experimental doses are higher than typical environmentally relevant concentrations and are therefore more suitable for identifying mechanisms than for the direct derivation of safety thresholds.

Two issues should be considered when interpreting these results. First, the effective concentration of particles or ions during germination is affected by seed-coat permeability, the soaking solution pH, phosphate level, and particle aggregation state. The relationship between nominal concentration and effective exposure has not been standardized, which limits cross-study comparisons. Second, dissolved Ce^3+^ may contribute substantially during germination, especially when particles undergo local dissolution, the exposure solution becomes acidic, or complexing ligands are present. However, because CeO_2_-NPs themselves have low solubility, germination toxicity should not be assumed to arise mainly from Ce^3+^. Effects observed during germination should be interpreted together with dissolution-supernatant controls, CeCl_3_ controls, and speciation analysis.

### 5.2. Root Growth and Architecture: Condition-Dependent Dose Responses to CeO_2_-NPs

**Figure 5:**
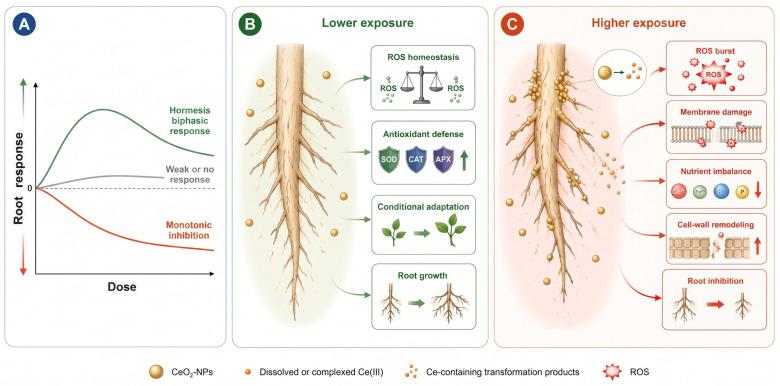
Condition-Dependent Root Responses to CeO_2_-NP Exposure. (**A**) Species- and system-dependent root growth responses; (**B**) adaptive stimulation at lower effective exposure; (**C**) oxidative injury and growth inhibition at higher effective exposure. Arrows indicate increasing exposure and the transition from adaptation to toxicity.

Root-growth responses to CeO_2_-NPs depend on exposure dose, particle properties, plant species, developmental stage, and exposure-medium chemistry. Available evidence does not support a universal biphasic or hormetic response. In a comparative root-elongation assay, CeO_2_-NPs produced no clear concentration-dependent response in wheat (*Triticum aestivum*) or rapeseed (*Brassica napus*) [1]. By contrast, CeO_2_-NPs promoted root elongation in cucumber and showed a weak inverted-U-shaped trend in maize (*Zea mays*), whereas *Lactuca* species exhibited root-elongation inhibition accompanied by ROS accumulation and membrane damage [18,45]. In soil-grown lettuce (*Lactuca sativa*), lower exposure was associated with growth promotion, whereas higher exposure reduced biomass and disturbed antioxidant homeostasis [17]. These contrasting outcomes indicate that CeO_2_-NP effects on root growth and architecture are species- and system-dependent, with non-monotonic responses occurring only under some exposure conditions (Figure 5A).

When stimulation occurs at lower exposure, it should be interpreted as a conditional adaptive response rather than as an intrinsic property of CeO_2_-NPs. Maintenance of ROS within a signaling-compatible range, together with moderate activation of endogenous antioxidant and metabolic defenses, may support temporary growth maintenance or stimulation under selected exposure conditions [9,10]. Changes in root-hair development, lateral-root formation, or hormone-associated responses may accompany this process, but their causal contribution remains insufficiently demonstrated in CeO_2_-NP–plant systems. Therefore, low-exposure stimulation should not automatically be classified as hormesis. The term hormesis should be used only when supported by adequate dose spacing, biological replication, and formal non-monotonic dose–response analysis (Figure 5B).

At higher effective exposure, accumulated CeO_2_-NPs and bioavailable Ce-containing transformation products can act as root stressors (Figure 5C). Adsorption and aggregation at the root surface, mucilage layer, and root tip can increase local particle–root interactions, while rhizosphere chemistry can redistribute Ce among particulate CeO_2_, dissolved or organically complexed Ce(III), and secondary phases such as CePO_4_ [2,32,33]. When local exposure exceeds the capacity of roots to maintain redox and mineral-nutrient homeostasis, ROS accumulation, lipid peroxidation, membrane injury, cell death, and nutrient-uptake disturbance can jointly inhibit root elongation, reduce root biomass, damage root tips, and alter root architecture [17,45,53]. The transition from apparent stimulation to injury should therefore be treated as a system-specific response boundary rather than as a universal property of CeO_2_-NPs.

### 5.3. Shoot Biomass and Leaf Morphology

Although CeO_2_-derived Ce is retained predominantly in roots, shoot biomass and morphology can still be affected by changes in root function, long-distance signaling, and low-level Ce transport. At low doses, stem height, leaf area, and shoot dry weight may increase slightly. This may be related to improved water and nutrient uptake by roots, the maintenance of photosynthetic activity, or altered growth-regulatory signals. At high doses, leaf chlorosis, reduced leaf area, leaf-edge curling, decreased chlorophyll content, and premature senescence may occur. These effects are often associated with impaired root uptake, mineral nutrient imbalance, enhanced oxidative stress, and ABA/ETH-related stress signals [1,36,44,54]. Unless IAA, ABA, ETH, or related transport- or synthesis-related genes are directly measured, shoot phenotypes should not be directly attributed to specific hormone pathways.

Shoot effects should not be regarded simply as a direct propagation of root effects. They may also arise through more complex indirect pathways. For example, changes in rhizosphere microbial functions may alter nitrogen and phosphorus supply and thereby affect shoot growth. Root ROS disturbances may also be transmitted to shoots through H_2_O_2_ waves, Ca^2+^ waves, or oxidized glutathione signals, thereby inducing defense responses. These indirect routes have not received sufficient attention in CeO_2_-NP–plant studies. Their causal status should be tested through time-series experiments and signal-blocking approaches. Thus, shoot phenotypes probably reflect a combination of root dysfunction, transported Ce species, nutrient imbalances, and systemic signaling, rather than a single direct toxicity pathway.

### 5.4. Photosynthetic System Responses and Stomatal Regulation

The photosynthetic system is an important target of CeO_2_-NP-induced stress under higher or prolonged exposure. It includes light harvesting by antenna complexes, primary photochemistry at the photosystem II (PSII) reaction center, the electron transport chain (ETR), and carbon assimilation through the Calvin cycle. These processes are interconnected in a cascade. A single photosynthetic parameter usually indicates functional disturbance but cannot independently identify the molecular target.

Typical responses include the following: (i) Decreased chlorophyll a/b content, which is more likely attributable to Mg/Fe nutritional disturbance, inhibited chlorophyll synthesis, or ROS-promoted degradation. There is insufficient evidence that Ce^3+^ directly substitutes for Mg^2+^ in the chlorophyll porphyrin center; therefore, this possibility should be described cautiously. (ii) Decreased maximum photochemical efficiency of PSII (Fv/Fm), indicating reduced PSII photochemical efficiency or increased photoinhibition. D1 protein damage should be discussed only when supported by protein-level evidence. (iii) Decreased photochemical quenching (qP) and increased non-photochemical quenching (NPQ), suggesting lower photochemical utilization and greater heat dissipation. However, increased NPQ can also be a protective response and should be interpreted together with ETR, Pn, Rubisco activity, and ROS indicators before concluding that carbon assimilation is limited. (iv) Suppressed Rubisco activity, possibly due to oxidative modification, insufficient nitrogen supply, or impaired Rubisco activase function. (v) Decreased stomatal conductance (gs) and transpiration rate, possibly related to water status, ABA-related signaling, and guard-cell function [54].

Under low-dose conditions, Ce^3+^/Ce^4+^ cycling within CeO_2_-NPs may indirectly protect the photosynthetic system by reducing overall oxidative pressure or buffering local ROS, thereby maintaining or slightly increasing Fv/Fm. Direct ROS scavenging near the photosynthetic electron transport chain should be discussed only when particles or transformation products are shown to reach chloroplasts, thylakoid membranes, or the electron transport chain. Most relevant reports derive from short-term hydroponic systems, and their reproducibility under long-term soil exposure remains to be tested.

### 5.5. Mineral Nutrient Uptake and Ionic Homeostasis

The effects of CeO_2_-NPs and bioavailable Ce-containing species on mineral nutrition are often underestimated in toxicity studies. Ce^3+^ and Ca^2+^ have similar ionic radii (approximately 1.01 vs. 1.00 Å), providing a chemical basis for possible competition at some binding sites or during certain transport processes. However, differences in valence, hydrated radius, and coordination preference indicate that the use of shared channels cannot be inferred solely from similarities in ionic radius. Ce^3+^ may disturb Ca^2+^ signaling, cytoskeletal stability, and mitosis [17,33,44]. CNGC, GLR, calmodulin, and CDPKs can be considered candidate targets, but evidence from direct binding, mutant studies, or functional blockade is required. High Ce exposure may also suppress Fe, Mn, and Zn uptake by roots. Possible mechanisms include competition between Ce^3+^ and Fe^2+^ for some metal transport systems, interference by root-surface Ce accumulation with FRO2-mediated reduction of Fe^3+^ to Fe^2+^, or physical obstruction of ion exchange by accumulated particle layers.

Mineral-nutrient imbalance may amplify CeO_2_-NP-induced stress. Iron deficiency can impair iron–sulfur proteins in the photosynthetic electron transport chain and aggravate photosynthetic inhibition. Disturbed Ca^2+^ signaling can affect stomatal opening and hormone signal transduction. Zinc deficiency may affect multiple zinc-finger transcription factors and SOD activity, thereby weakening antioxidant defense. This cascade from primary toxicity to nutrient imbalance and secondary amplification of toxicity may explain why whole-plant injury under high-dose exposure exceeds predictions based on a single mechanism.

### 5.6. Species-Specific Sensitivity and Comparative Analysis

Differences in plant sensitivity to CeO_2_-NPs are key variables in risk assessment (Table 3). Available studies suggest several conditional grouping patterns. However, these patterns should be regarded as empirical summaries based on limited plant materials and exposure systems rather than stable rankings of species sensitivity.

Monocot cereal crops, such as wheat, maize, and rice, show stimulation of root elongation and root-hair development in some low-dose CeO_2_-NP studies, and hormetic features appear relatively pronounced. The underlying mechanisms may involve differences in root structure, root exudates, and apoplastic barriers [1,2,49]. Legumes, including soybean (*Glycine max*), mung bean (*Vigna radiata*), and cowpea (*Vigna unguiculata*), have complex Fe/Mo nutritional networks associated with rhizobial symbiosis [49]. Because symbiotic nodulation is sensitive to redox conditions, legumes may be more sensitive to Ce^3+^ migration and redox disturbance. Legume seeds are also important nodes in food-chain transfer and are therefore relevant to food safety assessment [9,48,55]. Aquatic plant sensitivity should not be generalized. Small floating plants such as duckweed (*Lemna minor*) are in direct contact with the medium through both roots and fronds and have limited barrier structures and dilution capacity, which may make them more sensitive to particulate and ionic Ce. Aquatic plants with developed vascular tissues and aerenchyma, such as water hyacinth (*Eichhornia crassipes*), should be discussed separately [56,57]. Brassicaceous vegetables, such as radish (*Raphanus sativus*) and lettuce, show clear changes in photosynthetic parameters under soil exposure and may serve as sensitive indicator species for soil exposure risk [9,54]. Fruit vegetables, such as cucumber and tomato (*Solanum lycopersicum*), are useful model systems for studying rhizosphere transformations because their root exudates are abundant and can be precisely analyzed in hydroponic systems [2,32,33].

Species sensitivity is not controlled by a single factor. It reflects root architecture, exudate composition, the diversity of nutrient uptake proteins, endogenous ROS status, and basal expression of antioxidant enzyme genes. Therefore, dose–effect relationships established in a single species, such as cucumber or wheat, should be extrapolated with caution. Risk assessment should include multiple species representing different plant functional types.

**Table 3:** Response characteristics of representative plant species exposed to CeO_2_-NPs.

Plant Species	Functional Type	Exposure System and Dose Unit	Sensitive Endpoints	Dose–Response Trends Reported in the Literature	References
Wheat (*Triticum aestivum*)	Monocot	Hydroponic (mg/L)	Root length, root hair density, biomass	100–500 mg/L in hydroponics reported as stimulatory; >1000 mg/L reported as inhibitory	[1]
Maize (*Zea mays*)	Monocot	Hydroponic/foliar/soil (mg/L, mg/kg)	Root architecture, antioxidant enzymes	Low dose or foliar treatment reported as stimulatory; 2000 mg/kg soil exposure can show strong toxicity	[2,26,55,58]
Rice (*Oryza sativa*)	Monocot	Soil (mg/kg)	Ce accumulation, rhizosphere microbiome	100 mg/kg in soil reported as stimulatory; 500 mg/kg reported as inhibitory	[59]
Cucumber (*Cucumis sativus*)	Dicot fruit vegetable	Hydroponic/soil (mg/L, mg/kg)	Rhizosphere transformation, Ce speciation, transport	High hydroponic doses may promote root/stem growth but reduce germination; approximately 10 mg/kg in soil reported as favorable	[2,15,17,39,44]
Tomato (*Solanum lycopersicum*)	Dicot fruit vegetable	Hydroponic/soil (mg/L, mg/kg)	Fruit yield, biomass, H_2_O_2_, fruit Ce accumulation	0.1–10 mg/L reported as favorable in some studies; higher hydroponic doses or 50 mg/kg soil exposure may cause negative effects	[48,55]
Radish (*Raphanus sativus*)	Brassicaceous vegetable	Soil (mg/kg)	SPAD, photosynthetic parameters	50 mg/kg reported as stimulatory; >500–1000 mg/kg reported as damaging photosynthesis or growth	[60]
Lettuce (*Lactuca sativa*)	Leafy vegetable	Soil (mg/kg)	Root growth inhibition, CeO_2_ → Ce^3+^ transformation in roots, oxidative damage, biomass loss	100 mg/kg reported as stimulatory; >500–1000 mg/kg reported as inhibitory and possibly associated with oxidative damage	[9]
Arabidopsis (*Arabidopsis thaliana*)	Model plant	Hydroponic (mg/L)	Transcriptome, hormone signaling	≤250–500 mg/L reported as stimulatory in some studies; ≥1000 mg/L more often negative	[9,61]
Duckweed (*Lemna minor*)	Aquatic plant	Hydroponic (mg/L)	Thresholds for particulate and ionic toxicity	Aquatic systems such as duckweed often show lower thresholds; exact inhibition levels depend on Ce form and exposure design	[62,63]

Note: Reported responses are study-specific. Differences in exposure media, dose units, particle properties, exposure duration, developmental stages, and endpoints preclude direct comparison of dose thresholds across plant species.

In summary, plant morphological and physiological responses to CeO_2_-NPs depend strongly on effective exposure, plant species, developmental stage, particle properties, and exposure medium.

## 6. Redox Homeostasis, Metabolic Reprogramming, and Molecular Responses

### 6.1. Condition-Dependent ROS Homeostasis Under CeO_2_-NP Exposure

Reactive oxygen species (ROS) act as signaling molecules at controlled levels but cause oxidative injury when their production exceeds cellular buffering capacity. CeO_2_-NPs should therefore not be classified a priori as either antioxidants or pro-oxidants. Their net effects on plant ROS homeostasis depend on effective exposure rather than nominal concentration alone and are influenced by particle size and aggregation state, surface Ce^3+^/Ce^4+^ ratio, oxygen-vacancy density, coating chemistry, rhizosphere transformation, tissue localization, exposure duration, and the plant’s basal antioxidant capacity (Figure 5B,C) [9,10,13,14,15,16]. This Ce-specific redox framework should not be generalized to other REE-NMs.

In selected low-exposure or stress-mitigation systems, CeO_2_-NP treatment has been associated with lower ROS accumulation, reduced lipid peroxidation, or maintenance of antioxidant function [64,65,66]. These outcomes are consistent with a net ROS-buffering effect but do not, by themselves, demonstrate direct intracellular nanozyme activity. Establishing in planta Ce^3+^/Ce^4+^ catalytic cycling would require convergent evidence that CeO_2_ is present at the relevant subcellular site, retains a catalytically competent surface state, alters defined ROS in a temporally resolved manner, and produces effects distinguishable from endogenous antioxidant responses and from dissolved or complexed Ce species. Because most studies satisfy only part of these criteria, “ROS buffering” should be used as a phenotypic description unless direct mechanistic evidence is available.

With increasing effective exposure, the balance may shift toward oxidative stress. Root-surface accumulation, aggregation, sedimentation, and rhizosphere or tissue transformation can alter local exposure to particulate CeO_2_ and Ce-containing transformation products. Surface redox reactions may occur under specific pH, ligand, substrate, and coating conditions [13,14,15,16,43]. In parallel, bioavailable Ce(III)-containing species may disturb mineral-nutrient and metalloenzyme homeostasis, while particle or transformation-product accumulation at cell-wall and membrane interfaces may promote lipid peroxidation, membrane dysfunction, and antioxidant–enzyme imbalance [17,45,53,67]. Direct evidence that intact CeO_2_-NPs enter chloroplasts or mitochondria and exert primary redox effects at these sites remains limited; organelle-specific mechanisms should therefore be interpreted cautiously.

When ROS production exceeds the combined capacity of SOD, CAT, POD, APX, and the AsA–GSH cycle, reversible redox regulation gives way to oxidative injury. This transition is reflected by increased MDA and electrolyte leakage, loss of membrane integrity, cell death, and impairment of root or photosynthetic function (Figure 5C) [17,45,53,67]. However, the exposure level at which this transition occurs should not be treated as a universal threshold. It varies with plant species, medium chemistry, particle aging, exposure duration, and the relative proportions of particulate CeO_2_, dissolved or complexed Ce(III), and secondary Ce phases. Because current evidence is dominated by germination tests, hydroponic studies, and short-term soil experiments [9,10], defining this response boundary in continuous soil–rhizosphere–plant systems remains an important research priority.

### 6.2. Dose-Dependent Responses of Antioxidant Enzymes

The antioxidant enzyme system of plants often shows dose- and time-dependent responses to CeO_2_-NP exposure. An initial increase followed by a decline is a common response, but it is not the only pattern. Low-to-moderate doses can induce coordinated increases in SOD, CAT, APX, and GR, forming an effective H_2_O_2_-scavenging chain [68]. SOD converts O_2_•^−^ to H_2_O_2_, and CAT/APX convert H_2_O_2_ to H_2_O. Under high doses or long-term exposure, antioxidant enzyme activity may decline due to oxidative damage to enzyme proteins, displacement of metal cofactors at active sites, disrupted post-translational modification, or suppressed gene expression [17,18,33,44]. Different enzymes, tissues, and sampling times may show contrasting trends. ROS, MDA, AsA–GSH status, and growth endpoints should therefore be interpreted together [56].

POD responses are relatively complex. Abnormally high POD activity at moderate doses may indicate the simultaneous activation of cell-wall reinforcement processes, such as lignin synthesis, and defensive phenolic metabolism. APX is a major enzyme involved in H_2_O_2_ removal in chloroplasts, and its activity is closely related to photosynthetic injury. APX activity is therefore a sensitive biochemical indicator of photosynthetic stress.

Cross-study comparisons of antioxidant enzyme activity are affected by methodological differences. Enzyme assays, sampling times, tissue partitioning (whole root, root tip, or leaf), and exposure media can all cause substantial variation. Inferring protection or toxicity solely from increases or decreases in enzyme activity, without considering the experimental context, is a common oversimplification in the literature.

### 6.3. Non-Enzymatic Antioxidants and the AsA–GSH Cycle

The AsA–GSH cycle (ascorbate–glutathione cycle) is a major non-enzymatic and enzymatic pathway for H_2_O_2_ removal in chloroplasts and the cytosol. It operates through APX, MDHAR, DHAR, and GR. Under CeO_2_-NP exposure, changes in key nodes of this cycle can indicate the overall cellular redox status. A lower GSH/GSSG ratio suggests redox imbalance. Reduced AsA content indicates depletion of antioxidant buffering capacity. Increased GR activity suggests enhanced regeneration of the GSH pool [44,69].

Secondary antioxidant molecules, such as phenolics, flavonoids, and carotenoids, may also participate in plant responses to CeO_2_-NP exposure. Flavonoids can directly scavenge free radicals and complex metals. However, direct evidence is required to determine whether they substantially complex Ce^3+^ in plants and thereby reduce toxicity. Carotenoids, especially β-carotene and zeaxanthin, quench singlet oxygen (^1^O_2_) in the photosynthetic system, and changes in their levels are closely associated with photoprotection and photodamage. Phenylalanine ammonia-lyase (PAL) activity is commonly used as an indicator of phenylpropanoid activation. Its response pattern may also be dose- and time-dependent.

### 6.4. Lipid Peroxidation and Cellular Structural Damage

Lipid peroxidation is a biochemical marker of oxidative injury under higher CeO_2_-NP exposure. It is typically indicated by increased malondialdehyde (MDA) content. Polyunsaturated fatty acids (PUFAs) undergo peroxidative degradation through •OH-mediated chain reactions, and MDA can further form covalent cross-links with lysine residues in proteins, thereby impairing the functions of membrane-associated proteins [2,59,60,67]. Increased electrolyte leakage directly reflects the loss of plasma membrane integrity and serves as an early warning signal of cell death.

Transmission electron microscopy (TEM) studies have reported ultrastructural damage after high-dose exposure, including disruption of chloroplast thylakoid stacks, reduced mitochondrial cristae or increased matrix electron density, tonoplast rupture, and abnormal nucleolar morphology. These ultrastructural changes can serve as morphological evidence of oxidative stress and disturbances in energy metabolism, thereby supporting biochemical findings. However, sample preparation, sectioning position, and imaging field strongly affect observations. Systematic ultrastructural data remain limited, and these features should not be treated as universal markers of high-dose REE-NM exposure [70].

### 6.5. Primary Metabolic Reorganization: Carbohydrates, Amino Acids, and the TCA Cycle

Metabolomics provides a global view of how CeO_2_-NPs affect plant metabolic status.

Current studies show that metabolic changes induced by CeO_2_-NP exposure mainly involve carbon metabolism, the TCA cycle, and amino acid metabolism [2,33].

In carbon metabolism, changes in glucose, fructose, and sucrose concentrations reflect alterations in glycolysis and in the activities of sucrose synthase and sucrose-phosphate synthase. Trehalose accumulation is considered a marker of stress-related metabolic reorganization because it can contribute to osmotic adjustment and membrane protection.

In the TCA cycle, changes in citrate, α-ketoglutarate, malate, and succinate can indicate altered mitochondrial TCA flux. Citrate and malate are also major components of root exudates. Their intracellular accumulation may be linked to feedback involving root exudation and rhizosphere Ce^3+^ complexation, suggesting bidirectional coupling between cellular metabolism and rhizosphere chemistry.

In amino acid metabolism, proline accumulation is one of the most widely reported metabolic responses to osmotic stress and is often elevated under CeO_2_-NP treatment. GABA (γ-aminobutyric acid) is mainly produced from glutamate via glutamate decarboxylase (GAD), but it can also arise from polyamine degradation. It participates in pH regulation, carbon–nitrogen balance, and GABA signaling. Proline and GABA are both related to glutamate metabolism and stress responses, but they should not be regarded simply as upstream and downstream metabolites. Changes in branched-chain amino acids, including leucine, isoleucine, and valine, may reflect altered dynamics of protein synthesis and degradation.

At low doses, these metabolic changes often represent metabolic reprogramming that helps maintain energy balance and respond to redox disturbance. They resemble metabolic adaptation under mild stress. At high doses, inhibition of the TCA cycle, disruption of energy metabolism, and accelerated protein degradation indicate severe metabolic disruption and an increased risk of injury. Whether the metabolic system has entered an irreversible state requires evaluation through recovery experiments, cell death indicators, or later growth recovery data [71].

### 6.6. Secondary Metabolism: Phenylpropanoid and Sulfur Pathways

Activation of secondary metabolic pathways is an important defense response under CeO_2_-NP exposure. The phenylpropanoid pathway is often upregulated under stress and produces phenolic acids, flavonoids, and lignin precursors through the PAL–C4H–4CL enzyme cascade. These products contribute to antioxidant defense, cell-wall reinforcement, and metal ion complexation [44,69]. Phytochelatins (PCs) and metallothioneins (MTs) produced through sulfur metabolism may play roles in rare-earth ion chelation and detoxification similar to their roles in heavy-metal detoxification. However, plant PC/MT responses specifically associated with CeO_2_-NP exposure remain poorly studied.

### 6.7. Integrated Hormone Signaling Networks

Plant hormone signaling networks are important hubs for integrating CeO_2_-NP-associated redox, nutrient, and stress signals with physiological responses. Hormone responses may vary with dose. Low doses may affect IAA-related biosynthesis, transport, or response pathways and may be associated with root-tip cell elongation, lateral root formation, and root-hair differentiation [1,33,44,69]. Moderate doses may activate ABA-related signaling and mediate stomatal closure, the expression of osmotic adjustment genes, and enhanced seed dormancy. Accumulation of JA and SA often indicates systemic activation of defense-related secondary metabolism. Under high-dose conditions, ETH-related genes, such as those encoding ACC synthase and ACC oxidase, may be upregulated and associated with leaf chlorosis, premature root-tip senescence, and leaf abscission. These changes may occur together with disrupted polar IAA transport and sustained ABA accumulation. Hormone measurements, transcriptomic inferences, and phenotypic correlations constitute different levels of evidence. Correlations should not be interpreted as causal relationships. Direct causal evidence obtained using hormone-pathway mutants, biosynthesis or receptor inhibitors, and time-resolved hormone measurements remains limited in CeO_2_-NP–plant systems.

Hormone integration is not the simple additive action of individual pathways but instead occurs through hormone crosstalk. For example, JA and ABA can act synergistically to enhance osmotic stress responses. IAA and ETH can act antagonistically or synergistically in root elongation, root-hair formation, and lateral root development, depending on dose, tissue, and developmental stage. SA–JA antagonism regulates resource allocation between growth and defense. CeO_2_-NP-associated disturbance of hormone balance may amplify or buffer initial effects through these crosstalk networks, providing a possible basis for phenotypic differences among species under the same exposure conditions. Accordingly, hormone-related changes should be interpreted at present as an integrated response network associated with CeO_2_-NP exposure rather than as a set of fully established causal pathways.

### 6.8. Multi-Omics Integration and Mechanistic Modeling

**Figure 6:**
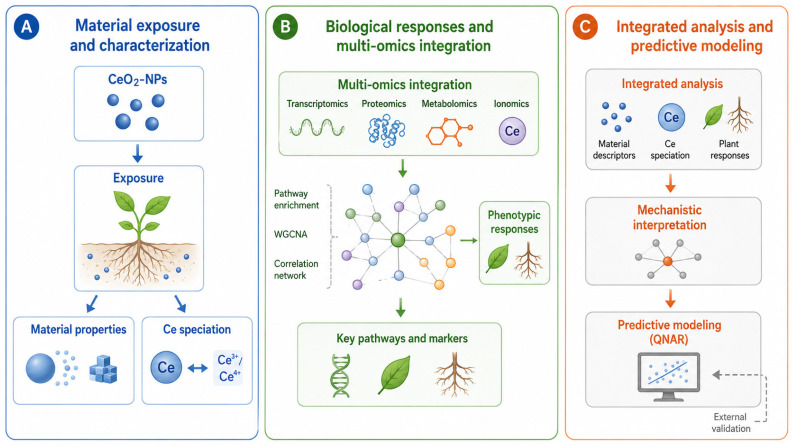
Multi-Omics and Elemental Speciation Framework for CeO_2_-NP Plant Responses. (**A**) Integration of multi-omics and Ce speciation data; (**B**) identification of key pathways and hub nodes; (**C**) development of QNAR models. Arrows indicate the workflow from multi-omics and elemental-speciation integration to network analysis and QNAR modeling.

Multi-omics integration is becoming an important approach for analyzing plant responses to CeO_2_-NPs (Figure 6A). Zhao et al. [33] combined transcriptomics and metabolomics in cucumber exposed to CeO_2_-NPs. In the 500 mg/L treatment group, upregulated genes were enriched in phenylpropanoid metabolism, glutathione metabolism, and MAPK signaling. Metabolomics showed increased levels of phenylalanine, tyrosine, proline, and GABA, whereas levels of TCA-cycle intermediates such as citrate and α-ketoglutarate decreased. Integrated analysis showed temporal correspondence between *PAL* gene upregulation and phenolic compound accumulation, suggesting transcriptional–metabolic coupling in the phenylpropanoid pathway. Further μ-XANES speciation analysis showed that root Ce occurred mainly as a mixture of CeO_2_ and CePO_4_, whereas shoots showed signals associated with Ce–organic acid complexes [33]. This multi-level evidence indicates that phosphate immobilization in roots and organic acid complexation during transport to shoots can occur simultaneously and may be spatially and temporally linked to the activation of secondary metabolic defenses.

Such systematic, integrative studies remain rare. Most studies use a single omics technology, or they measure multiple omics layers without cross-level integration [44,69]. The relationship between transcriptomics and proteomics, the causal link between metabolite changes and enzyme activity or gene expression, and the spatial coupling between elemental speciation and metabolic pathways remain major gaps in the current evidence. CeO_2_-NP effects should therefore not be interpreted on the basis of a single physiological indicator. Instead, interpretations should be supported by multi-level evidence chains linking material exposure, elemental speciation, molecular responses, and phenotypic outcomes.

At the data-integration level, pathway enrichment, weighted gene co-expression network analysis (WGCNA), and correlation network analysis can identify key response modules and candidate hub nodes from high-dimensional omics data (Figure 6B). These hub genes, proteins, or metabolites often show high connectivity and may lie at the intersections of ROS regulation, antioxidant defense, cell-wall remodeling, mineral nutrient homeostasis, and secondary metabolism. However, hub nodes primarily represent statistical centrality, not verified causal regulators. Their functions should be confirmed using qRT-PCR, protein abundance validation, enzyme assays, mutant or inhibitor experiments, or isotope tracing.

Current multi-omics studies of CeO_2_-NPs have clear limitations. Many studies focus on a single omics layer or a single endpoint. Exposure dose, material properties, plant species, growth media, and sampling times vary widely, making cross-study integration difficult. Simultaneous collection of particle physicochemical descriptors, elemental speciation data, transcriptome, proteome, metabolome, ionome, and phenotype data under unified exposure systems is a prerequisite for building quantitative nanostructure–activity relationship (QNAR) models (Figure 6C). When data volume and metadata standardization are sufficient, these models can test which material descriptors best explain variation in plant responses and provide reproducible statistical frameworks for cross-material and cross-species comparisons. At present, the available data remain limited. Model outputs are better treated as hypothesis-generating tools rather than as direct regulatory evidence.

In summary, changes in ROS homeostasis, antioxidant systems, primary and secondary metabolism, and hormone-associated signaling form an interconnected response network under CeO_2_-NP exposure. Oxidative stress and metabolic disruption are supported by consistent physiological and biochemical observations, but direct intracellular nanozyme activity and the causal roles of specific signaling nodes remain insufficiently validated. Multi-omics analyses are most informative when integrated with in situ elemental speciation, localization, and time-resolved phenotypic evidence.

## 7. Integrated Plant Toxicity Mechanisms: Dose Effects, Action Pathways, and Hydroponic–Soil Bridging

### 7.1. Non-Monotonic Dose–Response Curves and the Mechanistic Basis of Hormesis

Some REE-NM–plant–exposure systems show non-monotonic dose–response relationships. Low-dose ranges may show stimulation, whereas high-dose ranges show inhibition or toxicity, a phenomenon often referred to as hormesis [1,2,17,18,45,50,72,73]. Understanding this phenomenon requires more than phenotypic description. It also requires explaining why the same material can produce effects in opposite directions at different doses. Sufficient dose gradients and statistical models are also needed to confirm true hormesis rather than apparent biphasic responses caused by experimental noise or endpoint selection.

Available observations are consistent with several non-exclusive explanations. First, for CeO_2_-NPs, low doses may scavenge or buffer endogenous ROS generated by basal metabolism and maintain ROS within a range favorable for signaling. Higher doses may push ROS beyond this range and trigger oxidative damage. Second, low-level Ce^3+^ may produce weak stimulatory effects, but Ce is not recognized as an essential plant element and should not be equated with conventional mineral nutrients. High-dose Ce^3+^ may cause systemic metabolic disturbance through Ca^2+^ competition, metal transport interference, and nutrient antagonism. Third, low-dose particles may indirectly protect plants by adsorbing some coexisting toxic ions or altering the root-surface interface. This mechanism requires identification of specific ions and direct evidence and should currently be treated as a hypothesis.

Mechanistic interpretation of CeO_2_-NP-associated non-monotonic responses remains largely hypothetical. Time-resolved experiments that track ROS dynamics, Ca^2+^ signaling, hormone changes, and gene expression across doses remain limited. Future work should combine time-series analysis, recovery experiments, and dose–response modeling within the same system to distinguish true hormesis from short-term adaptation or apparent biphasic responses caused by endpoint selection.

### 7.2. Major Toxicity Pathways of CeO_2_-NPs and the Strength of Supporting Evidence

Based on current evidence, adverse plant responses to CeO_2_-NPs can be organized into five interacting pathways, although the strength of supporting evidence differs among them (Table 4) [9,10]. Oxidative stress, membrane injury, mineral-nutrient imbalance, and root-interface accumulation are supported by multiple physiological, biochemical, imaging, or speciation observations. By contrast, direct intracellular CeO_2_ nanozyme activity, the involvement of specific ion channels or transporters, causal hormone-signaling pathways, direct organelle targeting, and physical blockage of xylem vessels remain tentative. The relative contribution of each pathway depends on effective exposure and on the distribution of Ce among particulate CeO_2_, dissolved or organically complexed Ce(III), and secondary phases such as CePO_4_ [2,32,33,34].

Pathway 1 is oxidative stress and antioxidant-system failure. At higher effective exposure, root-surface accumulation, particle–root interfacial reactions, and bioavailable Ce(III)-containing species may increase net ROS production or weaken endogenous antioxidant defenses. When ROS generation exceeds the combined capacity of SOD, CAT, POD, APX, and the AsA–GSH cycle, accumulation of H_2_O_2_ and O_2_^−^, increased MDA and electrolyte leakage, and impairment of root or photosynthetic function may occur [17,45,53,67]. These endpoints support the occurrence of oxidative stress but do not, by themselves, demonstrate direct intracellular Ce^3+^/Ce^4+^ catalytic cycling. Protein carbonylation, DNA oxidation, or DNA strand breaks should be discussed only when supported by direct molecular assays.

Pathway 2 is membrane and subcellular injury. Excess ROS and Ce-containing species retained at cell-wall and plasma-membrane interfaces can promote lipid peroxidation, alter membrane permeability, and disturb cellular ion homeostasis [17,45,53,67]. Increased MDA and electrolyte leakage provide functional evidence of membrane injury. Reported alterations in chloroplast or mitochondrial structure, where available, may indicate secondary impairment of photosynthesis and energy metabolism, but ultrastructural observations should be interpreted together with functional measurements such as chlorophyll fluorescence, gas exchange, respiration, or membrane-potential analysis. Direct entry and action of intact CeO_2_-NPs within chloroplasts or mitochondria remain insufficiently demonstrated.

Pathway 3 is mineral-nutrient imbalance and disruption of ionic homeostasis. Root-surface Ce accumulation and bioavailable Ce(III)-containing species may alter the uptake or distribution of Ca, Fe, Mn, Zn, and P through changes in rhizosphere speciation, competition at binding or transport sites, membrane perturbation, or interference with root-surface ion exchange [17,19,55,56,74]. Because Ce(III) may occur as dissolved ions, organic complexes, adsorbed species, or secondary solid phases, total Ce concentration alone cannot identify the biologically active form. Specific channels or transporters should therefore be treated as candidate mechanisms unless supported by inhibitor experiments, genetic evidence, transporter-expression analysis, and concurrent Ce-speciation measurements.

Pathway 4 is hormone-associated signaling and growth regulation. Changes in IAA-, ABA-, or ETH-related molecular responses may accompany altered root growth, stomatal behavior, chlorosis, or senescence under CeO_2_-NP exposure [9,58]. However, most available evidence is based on transcriptomic associations, hormone measurements at limited time points, or correlations between hormone-related markers and phenotypes. Hormone signaling should therefore be interpreted primarily as a downstream integrated response to redox, nutrient, and membrane disturbances rather than as an established primary toxicity mechanism. Causal assignment requires time-resolved hormone profiling, pathway-specific mutants or inhibitors, and rescue experiments.

Pathway 5 is root-interface accumulation and transport interference. CeO_2_-NP aggregates and transformed Ce phases can accumulate at the root surface, within the mucilage layer, and in apoplastic spaces, thereby increasing local exposure and potentially restricting water or nutrient exchange [19,52]. Root-surface accumulation and apoplastic retention are more strongly supported than physical blockage of xylem vessels. Claims of xylem obstruction require direct imaging of vessel occlusion together with hydraulic-conductance or transport measurements; elemental localization near vascular tissues alone is insufficient [34]. Stomatal blockage associated with foliar application or atmospheric deposition should be evaluated separately and is not included in this rhizosphere-exposure framework.

These pathways are coupled rather than independent. Root-surface retention and transformation can modify local Ce speciation, while redox and mineral-nutrient disturbances can amplify membrane injury and hormone-associated responses, ultimately contributing to growth inhibition. Their relative importance varies with particle size, surface coating, aggregation and aging, rhizosphere chemistry, plant species, tissue localization, and exposure duration. Accordingly, the five-pathway framework should be interpreted as an evidence-graded synthesis of CeO_2_-NP phytotoxicity rather than as a fixed linear sequence or a universal model for other REE-NMs.

### 7.3. Evidence Positioning and Bridging Strategies Between Hydroponic and Soil Systems

Hydroponic and soil systems have distinct roles in CeO_2_-NP research, and their conclusions are not automatically equivalent. This remains an important methodological consideration.

Hydroponic systems allow precise control of exposure conditions, including particle concentration, pH, ionic strength, and nutrient-solution composition. The root exposure area can also be more readily defined. Root exudates can be collected in situ for speciation analysis. With synchrotron XAS, the chemical forms of Ce at root surfaces and within roots can also be characterized. Hydroponic systems are therefore useful for mechanistic investigation. Their limitations are also clear. They do not adequately capture soil-colloid adsorption, particle aging, organic-matter coating and regulation of aggregation, or realistic rhizosphere microbial communities. Aggregation, sedimentation, and root-surface coverage dynamics in hydroponic media also differ from those in soil pore water [1,2,32,33,34,45]. Hydroponic conclusions should therefore not be directly equated with conclusions regarding soil risk.

Soil systems have greater ecological relevance and provide more direct information for agricultural exposure scenarios. However, the high heterogeneity of soils, difficulty in controlling exposure doses, and complexity of monitoring rhizosphere chemistry limit mechanistic resolution. Artificial soil/sand culture, potted natural soil, and multi-year, multi-site field trials should be distinguished. The first two are useful for testing medium effects and rhizosphere processes. Multi-season field trials provide the highest level of extrapolative relevance.

Bridging hydroponic and soil systems requires improvements in both experimental design and data interpretation.

At the design level, hydroponic–soil bridging should be treated as a translation between evidence layers rather than as a simple concentration conversion. Feasible strategies include establishing medium gradients from hydroponics to sand/artificial soil and then to natural soil using the same material and plant species; simultaneously measuring total Ce, dissolved Ce, particulate Ce, pH, DOC, phosphate, and major cations in the nutrient solution or pore water; testing fresh and aged particles in parallel; and integrating root-surface adsorption, root internal accumulation, shoot translocation factor, and physiological endpoints into the same model. Medium type and aging time should be selected according to the research question, soil type, and expected exposure scenario. They should not be treated as fixed standards. Only after correspondence in concentration and speciation across multiple evidence levels is established can hydroponic mechanistic findings be more reliably extrapolated to long-term, low-dose soil exposure scenarios.

Field-scale evidence remains limited. A few multi-season field trials suggest that repeated application of CeO_2_-NPs may have cumulative effects on crop yield and soil microbial functions. However, the direction of these effects depends strongly on soil type, climate, background REE levels, and application mode [24]. A transferable framework for field risk assessment will require long-term, multi-site trials across climate zones and soil types, together with isotope labeling, metagenomics, pore-water speciation monitoring, and edible-part analysis. Such research should be a priority for the next stage of research.

In summary, the proposed toxicity pathways are interconnected and are not equally supported by experimental evidence. Oxidative stress, membrane injury, and nutrient imbalance are supported by multiple experimental endpoints, whereas direct transporter involvement, xylem blockage, and specific hormone-mediated causal pathways remain tentative. Reliable extrapolation from hydroponic systems to soils therefore requires matched characterization of exposure media, particle aging, chemical speciation, and plant responses.

## 8. Ecological Risk, Food Safety, and Environmental Management Implications

### 8.1. Rhizosphere and Endophytic Microbiomes and Soil–Plant Functions

Soil and plant-associated microbial communities are central to the ecological risk assessment of CeO_2_-NPs because they regulate nutrient cycling, plant nutrition, stress resistance, and nanoparticle transformation. Available evidence indicates that the effects of CeO_2_-NPs are spatially heterogeneous across the rhizosphere, rhizoplane, and internal root compartments and depend strongly on dose, exposure duration, soil conditions, coexisting elements, and plant-mediated changes in the root microenvironment.

A compartment-resolved study in soybean provided direct evidence that CeO_2_-NPs can alter both external and internal root-associated bacterial communities. Soybean plants were exposed to 1 or 100 mg kg^−1^ CeO_2_-NPs for either 84 or 190 days, and bacterial communities were analyzed separately in the rhizosphere, rhizoplane, and root tissue compartments. Exposure duration significantly affected bacterial α-diversity, with the 190-day treatment decreasing observed ASV richness and Shannon diversity, whereas both dose and duration contributed to changes in β-diversity [75]. The strongest compositional responses occurred in the rhizoplane under the low-dose, 84-day treatment, which showed the greatest number of differentially abundant ASVs. Changes were also detected in the root tissue compartment, indicating that CeO_2_-NP exposure can affect the endosphere-enriched bacterial community rather than only microorganisms in the surrounding soil. Predicted functional changes were dominated by biosynthesis and substrate degradation, utilization, and assimilation pathways rather than classical metal- or oxidative-stress pathways. Together with the strongest spatial response observed at the rhizoplane, these results suggest that altered plant physiology and root exudation mediated a substantial proportion of the bacterial response [75].

Rhizosphere responses are also strongly modified by soil geochemistry. In rice-planted soil exposed for 150 days to an environmentally relevant CeO_2_-NP concentration of 25 mg kg^−1^, CeO_2_-NPs altered bacterial community composition and soil metabolite profiles, although the nanoparticles alone did not significantly change α-diversity [76]. CeO_2_-NP exposure changed the relative abundances of Proteobacteria, Acidobacteria, Actinobacteria, Firmicutes, Bacteroidetes, Chloroflexi, Rokubacteria, Thaumarchaeota, and Nitrospirae. At the genus level, the abundance of the iron-reducing genus Geobacter increased, whereas the abundances of the plant-growth-promoting rhizobacterial genera Bacillus and Arthrobacter decreased. Ferrous amendment further modified these responses, reducing bacterial richness and diversity and altering the direction of several taxonomic and metabolic changes [77]. The associations among bacterial taxa, soil metabolites, enzyme activities, root Ce accumulation, and rice antioxidant responses indicate that microbiome composition, rhizosphere chemistry, and plant physiology form a coupled response system rather than representing independent endpoints.

A waterlogged Sb-contaminated rice system further demonstrated that CeO_2_-NP effects on rhizosphere bacteria cannot be generalized across environmental contexts. Individual CeO_2_-NP exposure caused little change in bacterial richness and diversity and produced community profiles similar to the control [61]. However, CeO_2_-NPs modified microbial responses to Sb exposure. In particular, CeO_2_-NPs alleviated some Sb-associated changes in Acidobacteria and reversed the inhibition of *Pseudomonas* abundance. The abundances of taxa associated with carbon, nitrogen, phosphorus, and metal transformation were correlated with rice growth, oxidative-stress indicators, and Sb accumulation [61]. These findings suggest that CeO_2_-NPs may disturb, buffer, or redirect rhizosphere community responses depending on soil redox conditions, contaminant speciation, and plant–soil interactions.

The relationship between CeO_2_-NPs and plant-associated microbiomes is bidirectional: microorganisms respond to CeO_2_-NPs but may also transform them. The root-associated beneficial bacterium *Pseudomonas* brassicacearum rapidly reduced Ce(IV) to Ce(III) when exposed to 4- and 7 nm CeO_2_ nanomaterials, whereas 31 nm particles remained largely untransformed [78]. The bacterial metabolites 2-ketogluconic acid and citric acid also induced partial Ce reduction, demonstrating that bacterial identity, metabolic activity, and particle size can regulate CeO_2_-NP speciation at the root–microbe interface [79]. Such microbial transformation may subsequently alter particle persistence, Ce bioavailability, and the biological effects experienced by both plants and microbial communities.

Not all beneficial bacteria are inhibited by CeO_2_-containing materials. A nanobiofertilizer study found no detectable inhibitory effect of the tested CeO_2_ nanocomposite concentrations on the micronutrient-solubilizing strains *Pseudomonas* gessardii and *Pseudomonas* azotoformans. The bacteria adhered to the CeO_2_ nanocomposite and remained functional in a formulation that promoted fenugreek (*Trigonella foenum-graecum*) germination and growth [80]. Although this study did not characterize whole-community composition, it demonstrates that CeO_2_–microbe interactions are strain- and formulation-specific and that selected beneficial microorganisms may remain compatible with CeO_2_-based agricultural formulations.

Microbiome responses should also be interpreted in relation to the chemical form of Ce encountered by roots and microorganisms. Smart radiolabeling experiments showed that short-term plant uptake and upward translocation of Ce following CeO_2_-NP exposure proceeded predominantly via a particulate pathway, with subsequent slower dissolution and redistribution occurring within plant tissues [65]. Therefore, changes in rhizosphere or endosphere-enriched bacterial communities should not automatically be attributed to dissolved Ce^3+^ toxicity. Intact particles, surface transformation, microbial metabolites, root exudates, and plant-mediated changes in rhizosphere chemistry may all contribute to these changes [65,79].

Overall, current evidence shows that CeO_2_-NPs can modify bacterial diversity and taxonomic composition in the rhizosphere, rhizoplane, and root tissue compartments, but the magnitude and direction of these effects depend on exposure history and environmental context. Future studies should combine compartment-resolved sampling of the rhizosphere, rhizoplane, surface-sterilized root endosphere, nodules, stems, and leaves with 16S rRNA and ITS sequencing, metagenomics or metatranscriptomics, root-exudate analysis, and in situ Ce speciation. Current evidence focuses primarily on bacterial communities, whereas fungal communities and strictly defined endophytic microbiomes remain substantially understudied.

### 8.2. Food-Chain Transfer and Food Safety Risk

Risk assessment of CeO_2_-NP-derived Ce entering the food chain should shift from a “total element accumulation” framework to one based on chemical speciation and bioaccessibility. Available studies have detected Ce signals above background levels in tomato fruits, soybean seeds, radish storage roots, and leafy vegetable edible parts [81]. However, most results represent total Ce levels and do not demonstrate that intact CeO_2_-NPs enter food tissues. Food safety assessment should at least distinguish among total Ce, dissolved Ce^3+^, particulate CeO_2_, CePO_4_, and organically complexed Ce [66]. It should also incorporate in vitro gastrointestinal digestion, bioaccessibility, intestinal epithelial absorption models, and dietary intake assessment. When these data are lacking, human health risk should not be inferred directly from crop accumulation. Cross-trophic transfer studies show that Ce can move from plants to herbivorous insects and soil animals. In the limited studies available, biomagnification factors (BMFs) are usually below 1, suggesting limited biomagnification potential in some terrestrial food chains. Nevertheless, limited biomagnification does not mean that risk is negligible. Sublethal effects, such as reduced reproduction, immune impairment, or behavioral changes, may occur in higher trophic organisms even without substantial Ce bioaccumulation and should be considered in multitrophic toxicology.

**Figure 7:**
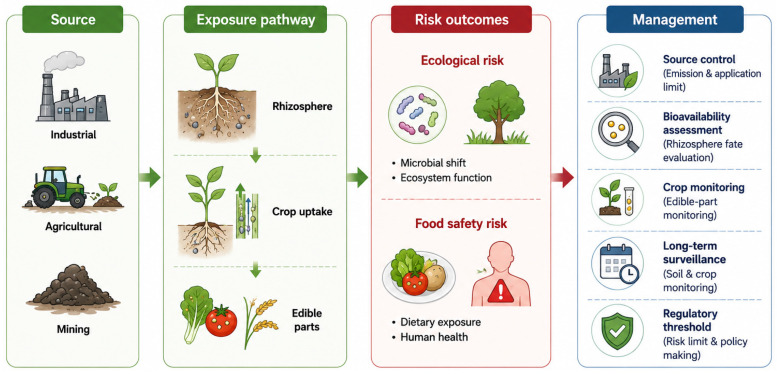
Exposure Pathways, Risks, and Management of CeO_2_-NPs in Soil–Crop–Food Chains.

The ecological risk of CeO_2_-NPs is not limited to toxicity at the individual plant level. It encompasses soil ecosystem services, crop uptake and accumulation, exposure of edible parts, food-chain transfer, and human dietary intake [82]. Figure 7 summarizes the potential risk chain of REE-NMs after their entry into the soil–crop–food chain system from four perspectives: sources, exposure pathways, risk outcomes, and management points. Rhizosphere transformation and total and speciated Ce accumulation in edible crop parts are key nodes connecting environmental exposure with food safety risk. Long-term monitoring, material-use regulations, scenario-specific application management, and edible-part analysis are key technical tools for future risk governance.

### 8.3. Human Health Risk and Regulatory Needs

Dietary intake of CeO_2_-NP-derived Ce is an important potential human exposure route. Health risk assessment should consider Ce concentration in edible parts, Ce chemical speciation, Ce bioaccessibility determined using in vitro digestion simulations, and intestinal absorption efficiency assessed using Caco-2 cell models. Bioaccessibility refers to the fraction released during gastrointestinal digestion that may become available for absorption. At present, quantitative human health risk data based directly on agricultural exposure scenarios remain very limited. Integrated exposure-assessment models covering field application, crop accumulation, dietary intake, gastrointestinal transformation, and intestinal absorption are urgently needed.

At the regulatory level, standardized databases for exposure, speciation analysis, and edible-part monitoring should first be established. Once sufficient evidence is available, application guidance and risk-management criteria for CeO_2_-based agricultural formulations could be developed. For rare-earth mining areas and potentially high-exposure farmland, long-term monitoring networks should be established to provide sustained data for regulatory decision-making.

In summary, ecological and food-safety risks of CeO_2_-NPs cannot be inferred from total Ce accumulation alone. Risk assessment should integrate rhizosphere transformation, chemical speciation, bioaccessibility, edible-part accumulation, and effects on soil microbial functions. However, field-scale exposure data, trophic transfer evidence, and quantitative information relevant to human dietary risk remain limited, and these evidence gaps underpin the research priorities outlined in Section 9.

## 9. Current Research Gaps and Future Directions

### 9.1. Key Evidence Gaps

From the mechanistic synthesis above, five gaps stand out as the most critical constraints on quantitative risk assessment of CeO_2_-NPs in soil–plant systems: (G1) direct causal linkage between rhizosphere transformation (CeO_2_ → Ce^3+^ → CePO_4_) and downstream root-uptake/translocation outcomes; (G2) quantitative resolution of particulate versus ionic contributions in planta; (G3) long-term, soil-based exposure data across representative crop species, especially over multiple growth cycles; (G4) species-level identification of the actual transporters mediating Ce^3+^ uptake and Ce^3+^-complex loading into xylem/phloem; (G5) causally interpretable integration of multi-omics evidence with in situ elemental-speciation and imaging data. The six priority research directions below are organised so that each direction addresses one or more of these gaps.

### 9.2. Need for Standardized Exposure and Characterization Systems

The most prominent bottleneck in CeO_2_-NP plant research is the lack of unified exposure designs and standards for characterization and reporting. This makes results from different laboratories difficult to compare and can generate apparently conflicting conclusions. Many of these inconsistencies may not reflect true biological differences but may instead arise from incomplete particle characterization, poorly reported exposure-medium chemistry, or fundamentally different plant culture conditions. This concern also extends to mixed-nanomaterial formulations and in vitro systems, in which the combination and dose ratio of SiO_2_NPs and CeO_2_NPs can alter the observed growth response [83].

Minimum reporting standards for nano-plant studies should be developed and promoted. Studies should report basic particle characterization, including TEM, DLS, ζ-potential, XRD, and XPS; exposure-medium pH, ionic strength, phosphate concentration, and organic matter content; particle stability, dissolution, and aging data in the exposure medium; plant species, cultivar, developmental stage, and environmental parameters; measured exposure concentrations in pore water or culture solution; and the relationship between experimental doses and predicted environmental concentrations or measured field concentrations, rather than reporting only nominal added concentrations. Because oxyanions and the concentration and valence of background electrolytes can alter CeO_2_-NP dissolution, aggregation kinetics, and colloidal stability [62,63,84], these medium-chemistry variables should be measured and reported explicitly.

### 9.3. Integrated Methods for Distinguishing Particulate CeO_2_ from Dissolved and Transformed Ce Species

Quantitatively resolving the distribution of Ce among particulate CeO_2_, dissolved or low-molecular-weight Ce species, organically complexed Ce(III), and secondary phases such as CePO_4_ remains one of the most important methodological challenges in CeO_2_-NP plant research [32,33,34,49,85]. A simple particulate-versus-ionic classification is insufficient because Ce(III) is not synonymous with freely dissolved Ce^3+^, and the detection of Ce(IV) does not independently demonstrate the persistence of intact CeO_2_ nanoparticles [32,33,34,49]. Total-Ce measurements should therefore be interpreted together with particle-sensitive, speciation-sensitive, and spatially resolved analyses.

No single analytical technique can quantitatively resolve all Ce pools. Matched experimental controls should include CeO_2_-NPs and soluble Ce salts at equivalent total-Ce concentrations to determine whether ionic Ce reproduces the uptake or biological responses observed under nanoparticle exposure [55,76]. Broader rare-earth-element controls may also help distinguish Ce-specific responses from more general rare-earth effects in sensitive model plants such as *Lemna minor* [86]. However, soluble Ce controls do not necessarily represent free Ce^3+^ activity because hydrolysis, organic-ligand complexation, adsorption, and phosphate precipitation may occur in nutrient solutions and rhizosphere environments [22,23,32,33]. Dissolution-supernatant controls, dialysis, ultrafiltration, or other operational size-fractionation approaches can further help distinguish particle-associated and low-molecular-weight fractions. Because these procedures may be affected by membrane adsorption, colloid breakthrough, and incomplete recovery, measurements of pH, phosphate, dissolved organic carbon, relevant organic ligands, and total-Ce mass balance should accompany particle–ion comparisons.

Single-particle ICP-MS (spICP-MS) can provide particle-sensitive information on particle number concentration and particle-equivalent size distribution, provided that tissue extraction preserves particle integrity and that recovery, aggregation, dissolution, and transformation artifacts are evaluated. Synchrotron XANES/EXAFS can constrain the Ce oxidation state and local coordination and, when suitable reference compounds are included, support the identification of CeO_2_, CePO_4_, and Ce–organic associations in plant tissues [32,33,34,49,85]. However, oxidation state alone cannot establish physical state. μ-XRF can provide spatially resolved elemental maps and identify Ce-enriched root, vascular, or leaf regions, but it cannot independently determine whether the detected Ce is present as intact CeO_2_, dissolved or complexed Ce, or a secondary Ce-containing phase [34,49]. TEM–EDS can provide complementary structural and elemental evidence for particle-associated Ce, although localized observations cannot independently quantify the dissolved contribution. Fluorescent or upconversion nanoprobe approaches have demonstrated the feasibility of tracing rare-earth nanomaterials or associated probes in living plants [78], while multimodal rare-earth nanoparticle imaging platforms developed in other biological systems illustrate possible complementary detection strategies [78]. Such optical or multimodal signals should nevertheless be treated as localization or tracking evidence and paired with Ce-specific particle and speciation analyses.

Isotopic or radiolabeling approaches can strengthen mass-balance and transport-kinetic analyses by tracing Ce derived from the original CeO_2_-NP treatment [61]. Nevertheless, radiolabeling does not independently demonstrate that Ce remains particle-associated after uptake and should therefore be combined with particle-sensitive and chemical-speciation measurements. Future studies should integrate these techniques within the same exposure system, sampling timeline, and tissue compartments to address four linked questions: (i) how much Ce remains particle-associated; (ii) whether, where, and to what extent CeO_2_ dissolution or transformation occurs; (iii) which Ce species reach different plant tissues; and (iv) how these species relate to biological responses. Convergent evidence from operational fractionation, particle analysis, chemical speciation, spatial imaging, radiolabeling, and total-Ce mass balance is required to distinguish particulate, dissolved or complexed, and secondary-phase contributions quantitatively.

### 9.4. Multi-Omics Integration and QNAR Modeling

At the mechanistic level, moving from single-endpoint measurements to integrated multi-omics represents an important methodological advance. Ideally, multi-omics studies should collect transcriptome, proteome, metabolome, ionome, and epigenome data within a unified exposure system, using the same particles, plant species, and exposure time points [86]. Reported changes in growth and antioxidant–enzyme activity following green-synthesized nanoceria exposure in tomato further emphasize the need to integrate physiological endpoints with molecular and speciation data [87,88]. Species-level differences in osmoprotectant and antioxidant responses under abiotic stress also show that generic stress-adaptation signatures should be distinguished from CeO_2_-NP-specific pathways [89]. WGCNA, pathway enrichment, causal inference, and network modeling can then be used to elucidate causal links across response levels and identify key regulatory nodes [33,44,59]. On this basis, combining particle physicochemical descriptors with intracellular speciation data to build QNAR models across species and media will be a key step in translating mechanistic understanding into toxicity prediction [90]. The reliability of QNAR models depends on standardized datasets of sufficient size, inclusion of negative results, external validation, and model interpretability. At this stage, QNAR is more suitable as a research framework and hypothesis-generating tool than as a mature regulatory prediction tool.

### 9.5. Long-Term Exposure, Transgenerational Effects, and Exposure Memory

Current research remains limited in its temporal scope. Most experiments last from days to weeks, whereas actual agricultural exposure to CeO_2_-NPs may continue for months or multiple growing seasons. Long-term exposure studies should examine the aging trajectories of particles in soil and the resulting effects on their bioavailability, plant adaptive responses under sustained low-dose exposure, and the transmission of phenotypic, transcriptomic, and epigenetic changes across generations [79,91]. Existing multigenerational studies of CeO_2_-NP exposure have reported transmissible physiological and biochemical changes in crop plants [79,91]. Plant exposure memory, whereby epigenetic marks induced by exposure in one generation are transmitted to the next and alter stress responses, has been reported under other types of stress. In the context of CeO_2_-NPs, this topic remains almost unexplored. Its existence and ecological significance require experimental validation.

### 9.6. Rhizosphere Triadic Coupling Among CeO_2_-NPs, Microorganisms, and Plants

Rhizosphere microorganisms play important roles in CeO_2_-NP fate and plant effects, but the three-way interactions among CeO_2_-NPs, microorganisms, and plants remain poorly studied. Future work should focus on quantifying the contributions of functional microbial groups, such as acid-producing bacteria, iron-reducing bacteria, nitrogen-fixing bacteria, phosphate-solubilizing bacteria, and AMF, to the rhizosphere transformation of CeO_2_-NPs. Other priorities include risk-mitigation experiments using synthetic communities (SynComs), investigating the adaptive evolution of rhizosphere microbial communities under long-term CeO_2_-NP exposure, and examining the dynamic coupling between microbially mediated Ce speciation processes—including potential Ce^4+^ reduction, Ce–organic matter complexation, CePO_4_ immobilization, and EPS adsorption—and plant uptake. These studies can deepen mechanistic understanding and support the development of microbial strategies for mitigating CeO_2_-NP-associated risks.

### 9.7. Safe-by-Design Strategies for Potential Agricultural Applications of CeO_2_-NPs

Beyond hazard characterization, CeO_2_-NPs intended for agricultural use should be designed and evaluated according to safe-by-design principles from the earliest stage of material development [92]. This approach should integrate mechanistic knowledge of nanoparticle toxicity and redesign, environmental fate, and plant cyto- and genotoxicity [92]. The objective should not be to maximize redox-modulating activity in isolation but to achieve a clearly defined agronomic function at the lowest effective exposure while minimizing root-surface overloading, formation of bioavailable Ce(III)-containing species, uncontrolled mobility and shoot translocation, long-term soil persistence, accumulation in edible tissues, and effects on non-target organisms. Relevant design variables include primary and hydrodynamic size, aggregation behavior, surface charge, coating identity and stability, surface Ce^3+^/Ce^4+^ ratio, oxygen-vacancy density, and dissolution kinetics [15,19,43,80]. Because these properties can change after contact with nutrient solutions, root exudates, phosphate, organic matter, and soil minerals, both the as-synthesized material and its environmentally transformed forms should be characterized [20,21,22,23,24,80].

Safe-by-design optimization must explicitly evaluate trade-offs among intended function, transformation, mobility, persistence, and biological effects. A coating that reduces acute particle–membrane interactions may improve colloidal stability and thereby increase particle mobility and the spatial extent of exposure. Conversely, a formulation that suppresses dissolution may reduce short-term exposure to dissolved or complexed Ce species but increase the persistence of particulate CeO_2_ in soil. Increasing surface Ce^3+^ abundance or oxygen-vacancy density may enhance intended redox-modulating performance under some conditions, but the direction and magnitude of ROS regulation can change with pH, ligand composition, phosphate availability, substrate concentration, and particle aging [15,19,20,21,22,23,24,80]. These properties should therefore be optimized jointly rather than independently.

Candidate formulations should be compared using matched fresh and aged particles across hydroponic, artificial-soil, and natural-soil systems [20,23,24]. Evaluation should include total Ce, particulate CeO_2_, dissolved or low-molecular-weight Ce species, organically complexed Ce(III), secondary phases such as CePO_4_, root retention, shoot and edible-tissue accumulation, plant performance, and rhizosphere microbiome responses. Application-specific benefits should be assessed together with recovery experiments, long-term soil persistence, repeated-exposure effects, and potential multigenerational responses [79,90], rather than inferred from nominal concentration or short-term growth promotion alone.

Ce should not be presented as a conventional fertilizer component, and CeO_2_-NP-induced growth stimulation should not be interpreted as evidence that Ce is an essential plant nutrient [9,10]. Where CeO_2_-NPs are investigated as carriers or stress-mitigation agents, the effects of the CeO_2_ core, surface coating, and loaded active ingredient should be separated using appropriate core-only, coating-only, and cargo-only controls [19,43,80]. A formulation should be considered promising only when its intended benefit is reproducible under soil-relevant conditions and is not accompanied by unacceptable increases in environmental persistence, mobility, edible-tissue accumulation, food-chain transfer, or ecological effects.

These priorities are interdependent. Standardized characterization defines the actual exposure entity; speciation-resolved analysis identifies the biologically relevant Ce pools; long-term soil and transgenerational studies evaluate aging, persistence, and cumulative effects; and integrated soil–microbiome–plant experiments determine whether the intended function remains compatible with ecological and food-safety constraints. These evidence gaps, priority approaches, and expected outcomes are summarized in Table 5.

## 10. Conclusions

Current evidence indicates that the behavior and effects of CeO_2_-NPs in soil–plant systems are governed by the combined influence of material descriptors, rhizosphere transformation, plant species, and physiological status rather than by nominal exposure concentration alone. Primary particle size, hydrodynamic diameter, surface charge, surface Ce^3+^/Ce^4+^ ratio, oxygen-vacancy density, and coating chemistry influence aggregation, sedimentation, dissolution, complexation, phosphate-mediated immobilization, and redox transformation in the rhizosphere, thereby shaping effective root-surface exposure, root retention, vascular transport and redistribution, and tissue Ce speciation. The resulting Ce pools—including particulate CeO_2_, dissolved or organically complexed Ce(III), surface-associated Ce, and secondary phases such as CePO_4_—may differ in uptake, transport, localization, and biological activity. Treating these forms as a single total-Ce exposure therefore obscures both mechanistic interpretation and hazard assessment. Plant responses to CeO_2_-NPs are best characterized as condition-dependent and bidirectional. Alterations in ROS homeostasis are frequently reported across experimental systems, but direct attribution of these effects to in situ intracellular Ce^3+^/Ce^4+^ catalytic cycling remains insufficiently supported by experimental evidence.

Several major knowledge gaps remain. Direct causal links between rhizosphere Ce speciation and downstream bioavailability and biological responses are still poorly established, and the relative contributions of particulate CeO_2_, dissolved or organically complexed Ce(III), and secondary Ce phases have not been quantitatively resolved in planta. The specific transporters mediating the uptake and vascular loading of bioavailable Ce-containing species remain unverified. Current evidence is dominated by short-term hydroponic studies, limiting extrapolation to aged soils, field conditions, repeated exposure, and multigenerational scenarios. In addition, rhizosphere microbiome responses, plant multi-omics, physiological endpoints, and elemental-speciation data are rarely collected with sufficient spatial and temporal resolution within the same experimental system.

Future research should prioritize standardized exposure and characterization protocols; matched particle–ion and formulation controls; integrated analytical workflows combining operational fractionation, spICP-MS, XANES/EXAFS, μ-XRF, TEM–EDS, isotopic or radiolabeling approaches, total-Ce mass balance, and bioaccessibility assessment; long-term soil-based, field, recovery, and transgenerational studies; and coordinated microbiome, multi-omics, physiological, and Ce-speciation datasets. These evidence chains are also required to develop interpretable and externally validated QNAR and machine-learning models and to support application-specific safe-by-design evaluation. The framework developed in this review is explicitly CeO_2_-centered. Although aggregation, root-surface adsorption, barrier retention, transport behavior, and condition-dependent dose responses may provide a basis for bounded comparisons across rare-earth nanomaterials, surface Ce^3+^/Ce^4+^ cycling, oxygen-vacancy-associated reactivity, and Ce-specific transformation among particulate CeO_2_, Ce(III) complexes, and CePO_4_ should not be directly extrapolated to La_2_O_3_-NPs, Y_2_O_3_-NPs, or upconversion nanoparticles. Strengthening these evidence chains is essential for advancing from phenomenological observations to mechanistic understanding, quantitative ecological and food-safety assessment, and scientifically defensible agricultural application.

## Figures and Tables

**Table 1:** CeO_2_-NP physicochemical descriptors, transformation scenarios, and plant-response endpoints.

Material or Transformation Scenario	Main Mode of Action	Common Plant Response Endpoints	Key Reference(s)
CeO_2_-NPs	CeO_2_-specific SOD/CAT-like and pro-oxidant reactions may coexist, depending on interfacial conditions	Root-surface adsorption, root-tip accumulation, root length/root hairs, SOD/CAT/APX, MDA, H_2_O_2_, chlorophyll, Fv/Fm	[2,17,19,22]
PAA-nCeO_2_	Nanozyme-like activity may remain, but surface carboxyl groups can change site accessibility	Lower root Ce accumulation, higher shoot translocation factor, xylem transport, leaf Ce signals	[15]
CS-nCeO_2_	High root-surface loading may induce ROS stress; independent coating effects must be separated	Strong root-surface adsorption, root Ce enrichment, lower translocation factor, root-tip growth inhibition or oxidative stress	[15]
Citrate/PVP-CeO_2_-NPs	Mild ROS buffering or interfacial passivation; relatively low acute toxicity	Seed germination, root length, biomass, antioxidant enzyme activation, conditional low-dose growth stimulation	[23]
CeO_2_-NPs + phosphate (transformation scenario)	Phosphate occupation of surface sites may reduce nanozyme-like activity	Root-surface CePO_4_ immobilization, reduced shoot transport, P nutrition interaction, rhizosphere speciation transformation	[22,32,33]
CeO_2_-NPs + organic acids (transformation scenario)	ROS scavenging or pro-oxidant effects depend on dose, ligand, and complexation state	Ce-carboxylate formation, increased xylem transport, root exudate feedback, organic-acid changes in metabolomics	[21,32,34]

Note: Particle-size ranges and dissolution/release tendencies are generalized from the literature and should not be used as quantitative thresholds across systems. The Ce^3+^/Ce^4+^ ratio and oxygen vacancies mainly apply to CeO_2_-NPs; La_2_O_3_, Y_2_O_3_, and UCNPs should not be interpreted using the CeO_2_ redox mechanism.

**Table 2:** Rhizosphere-condition-driven speciation transformation of CeO_2_-NPs and changes in bioavailability.

Rhizosphere Condition	Dominant Transformation	Expected Effect on Bioavailability	Evidence Sources
Near-neutral pH	CeO_2_-NPs remain mainly particulate; minor surface Ce(IV) → Ce(III) reduction	Relatively low bioavailability; mainly root-surface adsorption and root retention	[2,39]
Weakly acidic pH	Acid-promoted surface dissolution and Ce^3+^ release with organic ligand complexation	May increase; soil adsorption capacity must be considered	[25,39,44]
Strongly acidic microenvironment	Enhanced CeO_2_ surface dissolution and Ce^3+^ release	May increase; ionic toxicity and oxidative stress risk may rise; real rhizosphere frequency should be defined	[16,25,39]
High phosphate	Released Ce^3+^ rapidly precipitates with PO_4_^3−^ or nucleates on surfaces	Bioavailability decreases; Ce is immobilized at the root surface/rhizosphere and shoot transport decreases	[39,44]
Low phosphate	Ce^3+^ is less easily precipitated by phosphate and more likely to complex with organic acids	May increase; regulated by Fe/Al oxides and organic matter in real soils	[44]
Organic-acid rich	Surface complexation, local reduction, and Ce^3+^ migration; some acids may also form low-solubility precipitates	Often enhances migration; depends on acid type, concentration, pH, phosphate, and Ca^2+^ bridging	[39,44]
Organic matter/humic substances rich	Organic matter stabilizes particles and complexes Ce^3+^; may inhibit aggregation or promote colloid transport	Dual effect: free Ce^3+^ toxicity decreases, but colloidal transport may increase	[25,44]
Oxic condition	Ce remains mainly as Ce(IV); CeO_2_ lattice is relatively stable	Bioavailability is relatively low; effects mainly involve particulate root-surface adsorption and local interactions	[2,39,48]
Short-term reducing condition	Ce(IV) is reduced to Ce(III); local CeO_2_ dissolution increases	Bioavailability may increase transiently; mobility and root uptake risk may rise	[48]
Long-term redox alternation	Ce^3+^ release during reduction and re-precipitation or immobilization during re-oxidation	Bioavailability shows pulse-like changes; short-term increase but possible long-term decline with aging and immobilization	[48]
Strong root-surface adsorption	Electrostatic adsorption, carboxyl/phosphate complexation, local surface reduction	Local root bioavailability increases, but long-distance transport is limited	[2,15,39]
High microbial activity in the rhizosphere	Microbial acidification, EPS adsorption, potential reduction, and P release/immobilization jointly regulate Ce species	Direction uncertain: may increase via acidification/complexation or decrease via phosphate/EPS immobilization	[48]

Note: CeO_2_-NP bioavailability in the rhizosphere should not be inferred from total Ce concentration alone. Ce valence, particulate/ionic proportions, complexation state, precipitation/immobilization, spatial distribution, and evidence type should be considered. Low, moderate, and high are qualitative summaries and are not quantitative thresholds across systems.

**Table 4:** Proposed CeO_2_-NP toxicity pathways in plants and the strength of supporting evidence.

Pathway	Main Supporting Observations	Evidence Position	Major Limitation
Oxidative stress and antioxidant-system failure	ROS accumulation, altered antioxidant enzymes, increased MDA and electrolyte leakage, impaired root or photosynthetic function	Adverse outcome comparatively well supported; initiating mechanism context-dependent	Direct intracellular Ce^3+^/Ce^4+^ catalytic cycling remains unproven
Membrane and subcellular injury	Lipid peroxidation, electrolyte leakage, membrane-integrity loss, selected ultrastructural alterations	Membrane injury supported; organelle-specific mechanism tentative	Direct localization and action of intact CeO_2_-NPs in chloroplasts or mitochondria are unresolved
Mineral-nutrient and ionic-homeostasis disruption	Changes in Ca, Fe, Mn, Zn, or P uptake and distribution; root-interface accumulation	Context-dependent support	Active Ce species and responsible transport proteins remain unidentified
Hormone-associated signaling and growth regulation	IAA-, ABA-, and ETH-related molecular or physiological changes	Tentative	Evidence is mainly correlative and lacks pathway-specific validation
Root-interface accumulation and transport interference	Root-surface aggregation, mucilage association, apoplastic retention	Root-interface accumulation supported; xylem blockage tentative	Direct vessel-occlusion and hydraulic evidence are lacking

**Table 5:** Evidence gaps, priority approaches, and expected outcomes for CeO_2_-NP research in soil–plant systems.

Evidence Gap	Priority Approach	Expected Outcome
Inconsistent exposure and characterization	Standardized reporting of material properties, medium chemistry, effective exposure, and particle aging	Improved cross-study comparability
Incomplete separation of Ce pools	Integrated fractionation, spICP-MS, XANES/EXAFS, imaging, and total-Ce mass balance	Quantitative distinction among particulate CeO_2_, dissolved/complexed Ce, and secondary phases
Weak linkage between speciation and biological response	Time-resolved integration of Ce speciation, localization, physiology, and multi-omics	Stronger causal exposure–response relationships
Limited long-term soil evidence	Multi-season studies using fresh and aged particles in representative soils and crops	Better evaluation of persistence, cumulative effects, and field relevance
Poorly resolved soil–microbiome–plant coupling	Compartment-resolved microbiome, root-exudate, and Ce-speciation analysis	Identification of microbial transformation and non-target ecological effects
Insufficient safe-by-design validation	Matched comparison of size, coating, surface charge, redox state, dissolution, and aging	Application-specific optimization of function–risk trade-offs

Note: The proposed approaches should be implemented within matched exposure systems to link material design with environmental transformation and biological outcomes.

## Data Availability

No new data were generated or analyzed in this study. This article is a review of previously published research; all data discussed are available in the original publications cited in the reference list.

## Abbreviations

The following abbreviations are used in this manuscript:

4CL	4-coumarate:CoA ligase
ABA	abscisic acid
ACC	1-aminocyclopropane-1-carboxylic acid
AF4	asymmetric flow field-flow fractionation
AMF	arbuscular mycorrhizal fungi
APX	ascorbate peroxidase
AsA	ascorbate (reduced form)
BMF	biomagnification factor
C4H	cinnamate 4-hydroxylase
CAT	catalase
CDPK	calcium-dependent protein kinase
CeO_2_-NPs	cerium dioxide nanoparticles
CNGC	cyclic nucleotide-gated channel
CS	chitosan
DHAR	dehydroascorbate reductase
DLS	dynamic light scattering
DOC	dissolved organic carbon
EDS	energy-dispersive X-ray spectroscopy
ENPs	engineered nanoparticles
EPS	extracellular polymeric substances
ETH	ethylene
ETR	electron transport rate
EXAFS	extended X-ray absorption fine structure
Fv/Fm	maximum quantum yield of PSII photochemistry
GABA	γ-aminobutyric acid
GAD	glutamate decarboxylase
GC-MS	gas chromatography–mass spectrometry
GLR	glutamate receptor-like channel
GR	glutathione reductase
gs	stomatal conductance
GSH/GSSG	reduced/oxidised glutathione
IAA	indole-3-acetic acid
ICP-MS	inductively coupled plasma–mass spectrometry
JA	jasmonic acid
LC-MS	liquid chromatography–mass spectrometry
LMWOA	low-molecular-weight organic acid
MDA	malondialdehyde
MDHAR	monodehydroascorbate reductase
MT	metallothionein
NanoSIMS	nanoscale secondary-ion mass spectrometry
NPQ	non-photochemical quenching
NRAMP	natural resistance-associated macrophage protein
PAA	poly(acrylic acid)
PAL	phenylalanine ammonia-lyase
PC	phytochelatin
PEI	polyethylenimine
Pn	net photosynthetic rate
POD	peroxidase
PSII	photosystem II
PUFA	polyunsaturated fatty acid
PVP	polyvinylpyrrolidone
qP	photochemical quenching coefficient
QNAR	quantitative nanostructure–activity relationship
RBOH	respiratory burst oxidase homologue
REE-NMs	rare-earth element nanomaterials
REEs	rare-earth elements
REO-NPs	rare-earth oxide nanoparticles
ROS	reactive oxygen species
SA	salicylic acid
SOD	superoxide dismutase
spICP-MS	single-particle ICP-MS
SynCom	synthetic microbial community
TCA	tricarboxylic acid
TEM	transmission electron microscopy
UCL	upconversion luminescence
UCNPs	upconversion nanoparticles
WGCNA	weighted gene co-expression network analysis
XANES	X-ray absorption near-edge structure
XPS	X-ray photoelectron spectroscopy
XRD	X-ray diffraction
ζ-potential	zeta potential
